# Palmitoylation of vacuole membrane protein 1 promotes small extracellular vesicle secretion via interaction with ALIX and influences intercellular communication

**DOI:** 10.1186/s12964-024-01529-6

**Published:** 2024-02-26

**Authors:** Mengyuan Qu, Xinyu Liu, Xiaotong Wang, Zili Li, Liquan Zhou, Honggang Li

**Affiliations:** 1 Institute of Reproductive Health, Tongji Medical College, Huazhong University of Science and Technology, 13 Hangkong Road, Wuhan, 430030 Hubei China; 2 Present Address: The Reproduction Medical Center, The Third Affiliated Hospital of Shenzhen University (Luohu Hospital), 47 Youyi Road, Shenzhen, 518000 Guangdong China; 3 Wuhan Huake Reproductive Medicine Hospital, Wuhan, China; 4 The Third Affiliated Hospital of Zhengzhou University, Zhengzhou, China

**Keywords:** Extracellular vesicles, Multivesicular bodies, Palmitoylation, Sertoli cells, Spermatogonial stem cell like cells

## Abstract

**Background:**

Small extracellular vesicles (EVs), exemplified by exosomes, mediate intercellular communication by transporting proteins, mRNAs, and miRNAs. Post-translational modifications are involved in controlling small EV secretion process. However, whether palmitoylation regulates small EV secretion, remains largely unexplored.

**Methods:**

Vacuole Membrane Protein 1 (VMP1) was testified to be S-palmitoylated by Palmitoylation assays. VMP1 mutant plasmids were constructed to screen out the exact palmitoylation sites. Small EVs were isolated, identified and compared between wild-type VMP1 or mutant VMP1 transfected cells. Electron microscope and immunofluorescence were used to detect multivesicular body (MVB) number and morphology change when VMP1 was mutated. Immunoprecipitation and Mass spectrum were adopted to identify the protein that interacted with palmitoylated VMP1, while knock down experiment was used to explore the function of targeted protein ALIX. Taking human Sertoli cells (SCs) and human spermatogonial stem cell like cells (SSCLCs) as a model of intercellular communication, SSCLC maintenance was detected by flow cytometry and qPCR at 12 days of differentiation. In vivo, mouse model was established by intraperitoneal injection with palmitoylation inhibitor, 2-bromopalmitate (2BP) for 3 months.

**Results:**

VMP1 was identified to be palmitoylated at cysteine 263,278 by ZDHHC3. Specifically, palmitoylation of VMP1 regulated its subcellular location and enhanced the amount of small EV secretion. Mutation of VMP1 palmitoylation sites interfered with the morphology and biogenesis of MVBs through suppressing intraluminal vesicle formation. Furthermore, inhibition of VMP1 palmitoylation impeded small EV secretion by affecting the interaction of VMP1 with ALIX, an accessory protein of the ESCRT machinery. Taking SCs and SSCLCs as a model of intercellular communication, we discovered VMP1 palmitoylation in SCs was vital to the growth status of SSCLCs in a co-culture system. Inhibition of VMP1 palmitoylation caused low self-maintenance, increased apoptosis, and decreased proliferation rate of SSCLCs. In vivo, intraperitoneal injection of 2BP inhibited VMP1 palmitoylation and exosomal marker expression in mouse testes, which were closely associated with the level of spermatogenic cell apoptosis and proliferation.

**Conclusions:**

Our study revealed a novel mechanism for small EV secretion regulated by VMP1 palmitoylation in Sertoli cells, and demonstrated its pivotal role in intercellular communication and SSC niche.

**Supplementary Information:**

The online version contains supplementary material available at 10.1186/s12964-024-01529-6.

## Background

Extracellular vesicles (EVs), especially nano-sized EVs are widely accepted as important messengers for cell-to-cell communication. EVs contain RNA, DNA, proteins, lipids and metabolites of different functions, and transmit signals when taken up by recipient cells [1]. Exosomes are small EVs of 30–150 nm in diameter that produced by endosome way [2, 3]. They develop from in-budding of early endosomes, which, in turn, forms multivesicular bodies (MVBs) that contain intraluminal vesicles (ILVs). MVBs fuse with either lysosome to be degraded or the plasma membrane (PM) to secrete ILVs as exosomes [4]. Precise control of EV secretion is critical for intercellular communication and stable micro-environment. However, the mechanism of exosome-like small EV (sEV) biogenesis has yet to be elucidated.

S-palmitoylation is a post-translational modification consisting of the reversible attachment of the 16-carbon palmitate to specific cysteine residues via a thioester bond [5]. Palmitoylation enhances protein hydrophobicity and affects protein conformation, subcellular localization, membrane association, interaction with other effectors [6]. Dysregulation of palmitoylation has been recognized as a key mechanism for numerous pathological processes [7–9]. Whether palmitoylation regulate sEV secretion is an intriguing question. A recent study showed palmitoylation was required for Dsg2 to promote sEV biogenesis through regulating the subcellular localization of lipid raft and endosomal proteins, while inhibition of Dsg2 palmitoylation by inhibitor 2-bromopalmitate (2-BP) or mutating Dsg2 palmitoylation sites disrupted sEV secretion process [10]. In addition, exosomal and endosomal proteins are enriched in palmitoylation targets, such as tetraspanins (e.g. CD9, CD81) and Rab7 [11–13]. Palmitoylation is required for tetraspanin clustering, interactions and functions, which are essential for exosome biogenesis [11, 14]. A palmitoyl-proteomic analysis in prostate cancer cells identified a three-fold higher percentage of palmitoylated proteins in EVs versus cells (13.2% vs. 4.3%), and 41% of total putative palmitoylated proteins were identified in EVs [15]. However, the molecular mechanism by which protein palmitoylation regulates sEV secretion remains largely unknown.

Vacuole membrane protein 1 (VMP1), a conserved multi-spanning membrane protein, mainly localized in endoplasmic reticulum (ER) [16], is also found to be expressed in PM [17], endosomes, autophagosomes, lysosomes [18, 19]. It was originally identified to promote intracellular vacuole formation in pancreatitis [20], and essential for autophagosome formation in mammals [21, 22]. Loss of VMP1 causes increased PI3P signaling and tight contacts of isolation membranes with ER, thus resulting in impaired fusion of autophagosome with lysosome and disrupted autophagic flux [16, 23]. Studies in Drosophila [24], Dictyostelium [25] and Chlamydomonas [26] suggest that VMP1 is involved in diverse processes such as protein secretion, endo- and exocytosis, protein trafficking, organelle biogenesis, osmoregulation, cytokinesis. VMP1 depletion in Hela cells leads to defective endosome trafficking and maturation, and also the accumulation of endosomes at the perinuclear area [27]. Coincidentally, proteomics identifies that VMP1 is a putative palmitoylated protein [28]. Based on VMP1 effect on endosome maturation and protein secretion, we hypothesize that VMP1 may affect MVB formation and sEV secretion.

Spermatogonial stem cells (SSCs) are the stem cells that support spermatogenesis process. Sertoli cells (SCs) secrete various cytokines that are required for SSC self-renewal and differentiation. Moreover, the sEVs released by SCs have emerged as vital components in intercellular communication between SC and SSC [29, 30]. Researchers found that inhibition of SC derived-sEVs reduced spermatogonial proliferation and also alter the differentiation of SSCs in vitro [31, 32]. The SC-SSC communication is a representative intercellular model that explains the role of sEVs in signal transduction. However, the molecular mechanism of signal transmits by sEVs in SCs has not been reported thoroughly. In this study, we aim to identify VMP1 palmitoylation and elucidate the underlying molecular mechanism by which VMP1 palmitoylation affects sEV biogenesis and secretion. By taking human SCs and SSCLCs co-culture as a cell-to-cell communication model, we also explore the regulatory role of VMP1 palmitoylation on SSC niche and intercellular communication.

## Methods

### Antibodies and reagents

The following primary antibodies were used: rabbit polyclonal anti-VMP1 (ab240887; Abcam; UK); mouse monoclonal anti-flag (AE005; Abclonal; China); rabbit polyclonal anti-Na ^+^ -K ^+^ -ATPase (ab76020; Abcam); rabbit polyclonal anti-EEA1 (ab2900; Abcam); rabbit monoclonal anti-Rab7 (ab137029; Abcam); rabbit polyclonal anti-Rab11 (#71–5300; invitrogen; USA); mouse monoclonal anti-CD63 (ab217345; Abcam; UK); rabbit polyclonal anti-Alix (12422–1-AP; Proteintech; China); rabbit polyclonal anti-Tsg101 (ab83; Abcam); mouse monoclonal anti-LAMP1 (sc-19992; Santa cruz; USA); mouse monoclonal anti-CHMP4 (sc-514869; Santa cruz; USA). 2-BP (#21604) was purchased from Sigma Aldrich. Baf A1(#54645) was purchased from Cell Signaling Technology; When indicated, the medium contained 50 μM 2-BP or 20 nM Baf A1.

### Cell culture

Human SCs were a gift from Professor Zuping He at The Key Laboratory of Model Animals and Stem Cell Biology, Hunan Normal University. The HEK293T cell line was purchased from Shanghai GeneChem (Shanghai, China) and cultured in DMEM medium (Gibco, Grand Island, NY, USA) supplemented with 10% fetal bovine serum (FBS) (Gibco) and 1% penicillin–streptomycin (Beyotime Biotechnology, Beijing, China). SCs were kept in DMEM/F-12 medium. hiPSC lines were maintained in mTeSR1 medium (STEMCELL Technologies) at 37 °C and 5% CO2. For hiPSCs differentiation into SSCLCs, cells were firstly seeded on Matrigel (Corning)-coated 12-well plates in mTeSR1 medium containing 10 μM ROCK inhibitor, and the medium was replaced by SSCLC induction medium on the second day when cells reached 80%-90% confluence. The components of SSCLC induction medium were based on previous studies [33].

### Plasmid construction, mutagenesis and transfection

WT-VMP1-flag was purchased from WZ Biosciences Inc. China. Mutations in the palmitoylation sites of VMP1 were generated by site-directed mutagenesis using the QuikChange mutagenesis kit (Stratagene, 200,523). All mutations were confirmed by sequencing the entire DNA. For lentiviral overexpression, cDNAs were cloned into pCDH-EF1-MCS-IRES-Puro (System Biosciences). For knockdown, shRNAs of each gene were designed and cloned into pLKO.1 (Addgene, 10,878). For lentiviral infections, HEK293T and SCs were infected with lentivirus in medium containing 8–10 mg/ml polybrene. Cells were selected against 1 mg/ml puromycin for at least 48 h and then used for the described experiments. For DNA or siRNA transfection, HEK293T cells and SCs were transfected using Lipofectamine 2000 (Invitrogen) with DNA or small interfering RNAs. 24 h after transfection, cells were cultured in exosome-depleted medium for 20 h and sEVs were isolated for further analysis.

### Palmitoylation assays

SCs, HEK293T and other cells transfected with the indicated plasmids or siRNAs were incubated in medium supplemented with 100 μM 17-octadecynoic acid (17-ODYA), an alkyne-containing palmitate analog (Cayman Chemical, 90,270) for 6–8 h. Cells were then washed with phosphate-buffered saline (PBS) and briefly sonicated in detergent-free lysis buffer. Membrane fractions were made by ultracentrifugation and then pellets were lysed in lysis buffer with 1% (v:v) Triton X-100. An aliquot (94 μl) of each cleared lysate was reacted with 6 μl of freshly premixed click chemistry reagent (final 100 μM biotin-azide, 1 mM Tris [2-carboxyethyl] phosphine, 100 μM Tris-[benzyltriazolylmethyl] amine, and 1 mM CuSO4) [34]. Reaction mixtures were incubated for 1 h at room temperature (RT), and then 2 μl of 0.5 M EDTA was added to terminate the reactions. Unreacted biotin was quenched by the addition of an excess of 1 M Tris, pH 8.0. Biotinylated (palmitoylated) proteins were then affinity isolated using streptavidin beads by incubation at 4 °C for 2 h. The beads were washed 3 times with PBS containing 0.5% Triton X-100 and treated with sample buffer for western blot.

### Western blot

Testis tissue, cells, and exosome fractions were lysed in Mammalian Protein Extraction Reagent (78501, Thermo Scientific) containing a protease inhibitor cocktail. The proteins were separated by 8–12% bisacrylamide gel electrophoresis and transferred to PVDF membranes (Millipore, Billerica, MA, USA). The proteins were reacted with primary antibodies followed by a horseradish peroxidase (HRP)-conjugated secondary antibody. Immunodetection was carried out with Chemiluminescent HRP substrate. Normalization was conducted by blotting the same samples with an antibody against β-actin or GAPDH.

### Immunofluorescence and confocal microscopy

For tissue, Testicular tissues were fixed in 4% paraformaldehyde overnight, embedded in paraffin, and sectioned at 5 μm thickness. For cells, cells were harvested at 80% confluency and the cell slides were fixed with 4% paraformaldehyde, permeabilized with 0.5% Triton X-100 for 10 min, and probed with primary antibodies diluted 1:100 in PBS overnight at 4 °C. After washing in PBS, the cells were incubated with fluorescent-conjugated secondary antibodies (488 and 594 nm) for 1 h (1:200). All samples were treated with DAPI dye for nuclear staining (358 nm). TUNEL staining of apoptotic cells was performed by using the One Step TUNEL Apoptosis Assay Kit (Beyotime Biotechnology, China) following the manufacturer’s instructions. Images were captured with a LSM 780 confocal scanning microscope (Zeiss, Germany), and analyzed by ZEN 2010 software, Image J, Adobe Photoshop. A total of 30–50 cells were analyzed per condition in a blind manner, and the experiment was performed independently three times.

### Surface biotinylation assay

Cells were washed with PBS and incubated in 1 mg/ml Sulfo-NHS-LC-Biotin (Thermo Fisher Scientific, 21335) in PBS for 30 min on ice. Free biotin was quenched by the addition of 100 mM glycine in PBS. Lysates were prepared in lysis buffer by sonication, centrifuged at 20,000 g for 20 min at 4°C, and protein concentration in the supernatants was determined. Volume and protein content were equally adjusted in all samples and 12.5% avidin beads (Thermo Fisher Scientific, 29202) were added to each sample. After incubation for 2 h at 4°C, the beads were collected by centrifugation, washed 3 times with 0.5% Triton X-100 in PBS, and the proteins were extracted in sample buffer. Collected proteins were analyzed by western blot.

### Co-immunoprecipitation (Co-IP)

Co-IP was performed using the Pierce Co-Immunoprecipitation Kit (26149, Thermo Scientific) according to the manufacturer’s instructions. Briefly, cells were lysed with lysis buffer (25 mM Tris, 150 mM NaCl, 1 mM EDTA, 1% NP-40, and 5% glycerinum, pH 7.4) for 10 min on ice. The lysates were cleared by centrifugation at 13,500 × g for 10 min at 4°C and then immunoprecipitated individually with anti-flag antibody, anti-VMP1 antibody or normal IgG. After the elution, the proteins were lysed in RIPA lysis buffer for the western blot or mass spectrometric analysis.

### Mass spectrometric analysis

After co-immunoprecipitation, equal amounts of proteins were loaded in 4–12% SDS-PAGE gels and stained with Coomassie blue G250 (BioRad). After staining, the bands higher than 10 kDa were excised into individual fractions. These fractions were then further excised into small pieces and placed into a 1.5 ml tube. Sample preparation used for Q-Exactive mass spectrometry was performed according to the standard protocol. After destaining and shrinking, the gel was treated with 20 mM DTT for protein reduction, followed by 50 mM iodoacetamide (IAA) treatment for alkylation. Protein digestions were performed with trypsin at 37 °C overnight and the digested proteins were then desalted for LC–MS/MS analysis. Proteins were identified using Protein Pilot 4.0TM software.

### Isolation of sEVs

Current isolation methods do not separate endosome-derived sEVs from non-endosomal sEVs. To isolate sEVs from the cellular supernatant, 80% confluent cells were rinsed with PBS and refreshed with DMEM containing 10% exosome-depleted FBS. After a 48-h incubation, an equivalent volume of culture medium conditioned by an equivalent number of cells was collected, and followed by sequential centrifugation. Briefly, the medium was centrifuged at 300 × g for 10 min to remove cells. The supernatant was centrifuged at 2000 × g for 10 min and then at 10,000 × g for 30 min. The pellets were collected and referred to as the 2 K pellet and the 10 K pellet, respectively. The resulting supernatant was filtered through a 0.22-μm filter, and sEVs were pelleted by ultracentrifugation at 100,000 × g for 70 min. The concentrations of sEV proteins were measured using a BCA Protein Assay Kit (Thermo Fisher) according to the manufacturer’s instructions. The final pellets were resuspended in ice-cold PBS.

### Transmission electron microscopy

A 10 μl aliquot of freshly isolated sEVs were allowed to dry on a formvar/carbon-coated copper grid for 10 min and were fixed in 3% glutaraldehyde for 10 min, rinsed in water, and contrasted in a uranyl acetate (4%)/methylcellulose (1%) mix for 10 min at room temperature to negatively stain the exosomal fractions. Then the samples were observed immediately at 80 kV with a JEOL-1200EX electron microscope. WT-VMP1 and MT-VMP1 SCs were rinsed in 0.1 M PBS and fixed with 2.5% glutaraldehyde overnight at 4 °C for the conventional electron microscopic analysis.

### Nanoparticle tracking analysis

The number and size of sEVs were directly tracked using the NS300 instrument (Malvern Instruments Ltd., Worcestershire, UK) equipped with a 488 nm laser and a high-sensitivity sCMOS camera. In this analysis, particles were automatically tracked and sized based on Brownian motion and the diffusion coefficient. The sEV pellets were resuspended and diluted in PBS to obtain a concentration within the recommended range and vortexed for 1 min. The samples were loaded into the sample chamber at ambient temperature. Three 30-s videos were acquired for each sample. The videos were subsequently analyzed with the NTA2.3 software.

### PKH26 labelling and co-culture experiment

Exosome-like sEV from SCs were labelled using a PKH26 red fluorescent labelling kit (Sigma) according to the manufacturer's protocol. Briefly, the sEVs were incubated with the PKH26 dye for 4 min, and the reaction was terminated by adding exosome-depleted FBS Media Supplement (SBI). Then, the sEVs were washed three times and excess PKH26 dye removed by 100 kD Amicon Ultra-4 (Millipore), then incubated with SSCLCs. The uptake of sEVs into cells was observed by confocal microscope. In the co-culture experiment, SCs were inoculated at a density of 1 × 10^5^/well in the upper chamber of a Transwell (0.4 μm), while the SSCLCs were in the lower chamber at a density of 1 × 10^5^/well. SCs were transfected with WT-VMP1-Flag, MT-VMP1-Flag and NC plasmid respectively in the upper layer. After 24 h of co-incubation with treated SCs, SSCLCs were harvested for experimental tests.

### Edu proliferation assay

SSCLCs were co-cultured with differently transfected SC (WT, MT, NC) groups, and the cell proliferation was detected through the incorporation of 5-ethynyl-29-deoxyuridine (EdU) with the EdU Cell Proliferation Assay Kit (Sangon, China). Briefly, the cells were incubated with 50 mM EdU for 3 h before fixation, permeabilization and EdU staining, which were performed according to the manufacturer’s protocol. The cell nuclei were stained with DAPI (Sigma) for 10 min. The proportion of cells that incorporated EdU was determined by fluorescence microscopy.

### Flow cytometry

Cells were dissociated by Accutase (Thermo Fisher Scientific), and fixed by 4% PFA for 20 min at room temperature. The fixed cells were suspended in fluorescence activated cell sorting (FACS) buffer (PBS with 5% fetal calf serum, 0.2% Triton-100, and 0.5% Tween 20) for 25 min and centrifuged at 500 X g for 5 min before incubation with a PLZF antibody (Invitrogen, Mags.21F7, 1:200 dilution). Subsequently, the cells were washed with FACS buffer, detected with PE-conjugated anti-mouse second antibody. Apoptosis analyses were performed using FITC-Annexin V Apoptosis Detection Kit from BD Pharmingen according to the manufacturer’s instructions.

### Establishment of animal model

Adult male C57/BL6 mice (6-week old) were purchased and bred in the Animal Center of Tongji Medical College. The animals were kept at 25 °C with a 12 h light/dark cycle and fed with standard food pellets and water ad libitum. Randomly selected mice (n = 10) were kept as the control group (CTR) and injected with saline solution. Another 20 mice were anesthetized and intraperitoneally daily injected with palmitoylation inhibitor 2BP (1 mg/kg) for three months (2-BP group) continuously. The mice of 2BP and CTR group were then anesthetized and sacrificed. We collected and processed their testes for immunofluorescence and immunoblotting. The study was approved by the Ethical Committee, Review Board of Tongji Medical College, Huazhong University of Science and Technology, China.

### Acyl-biotinyl exchange assay

Palmitoylation of tissue protein was assessed by IP and acyl-biotin exchange (IP-ABE) as previously described [35]. Briefly, protein from the testis tissue was extracted using a lysis buffer with protease inhibitors and N-ethylmaleimide. After precipitating VMP1 protein with VMP1 antibody (#12929; Cell Signaling Technology; USA) and magnetic beads (Millipore), samples were re-suspended with stringent buffer. Each sample was divided into two parts: two thirds of the lysis sample part were mixed with 500 μl 1.5 M NH_2_OH solution (Sigma-Aldrich) (+ NH_2_OH Sample), and the one third part was mixed with 500 μl 0.1 M Tris–HCl (pH 7.4) (-NH_2_OH Sample). All samples were rotated at room temperature for 50 min. Then samples were added with Biotin-BMCC buffer, and rotated for 50 min at 4°C. Proteins were eluted from the beads by boiling in sample buffer and the cleared supernatants were resolved by SDS-PAGE and electro-transferred to PVDF membranes, and then followed by western blotting.

### Quantitative real-time PCR

The total RNAs from SCs, SSCLCs or testis tissues were extracted with TRIzol reagent (Invitrogen, USA), and complementary DNA (cDNA) was synthesized with the ReverTra Ace qPCR RT Kit (Toyobo). Q-PCR was performed in triplicate with SYBR Green Realtime PCRMaster Mix (Toyobo). The primers of targeted genes were synthesized by Biosune (Shanghai, China). GAPDH was used to normalize mRNA expression. The expression was calculated by the 2^−ΔΔCT^ method.

### Statistical analysis

Data were expressed as the mean ± SEM. For normally distributed data, unpaired Student’s t-test was used for comparisons between two groups and ANOVA followed by Tukey multiple comparison test for comparisons among multiple groups. For data that were not normally distributed, Mann–Whitney rank-sum test was used for comparisons between two groups. Three independent times of each experiment were performed and statistically analyzed. All statistical analyses were performed using Graph Prism 8.0 software (GraphPad Software Inc. San Diego, USA). Statistical significance was considered if a *P*-value was < 0.05.

## Results

### VMP1 is palmitoylated at cysteine 263 and 278 by ZDHHC3

**Fig. 1:**
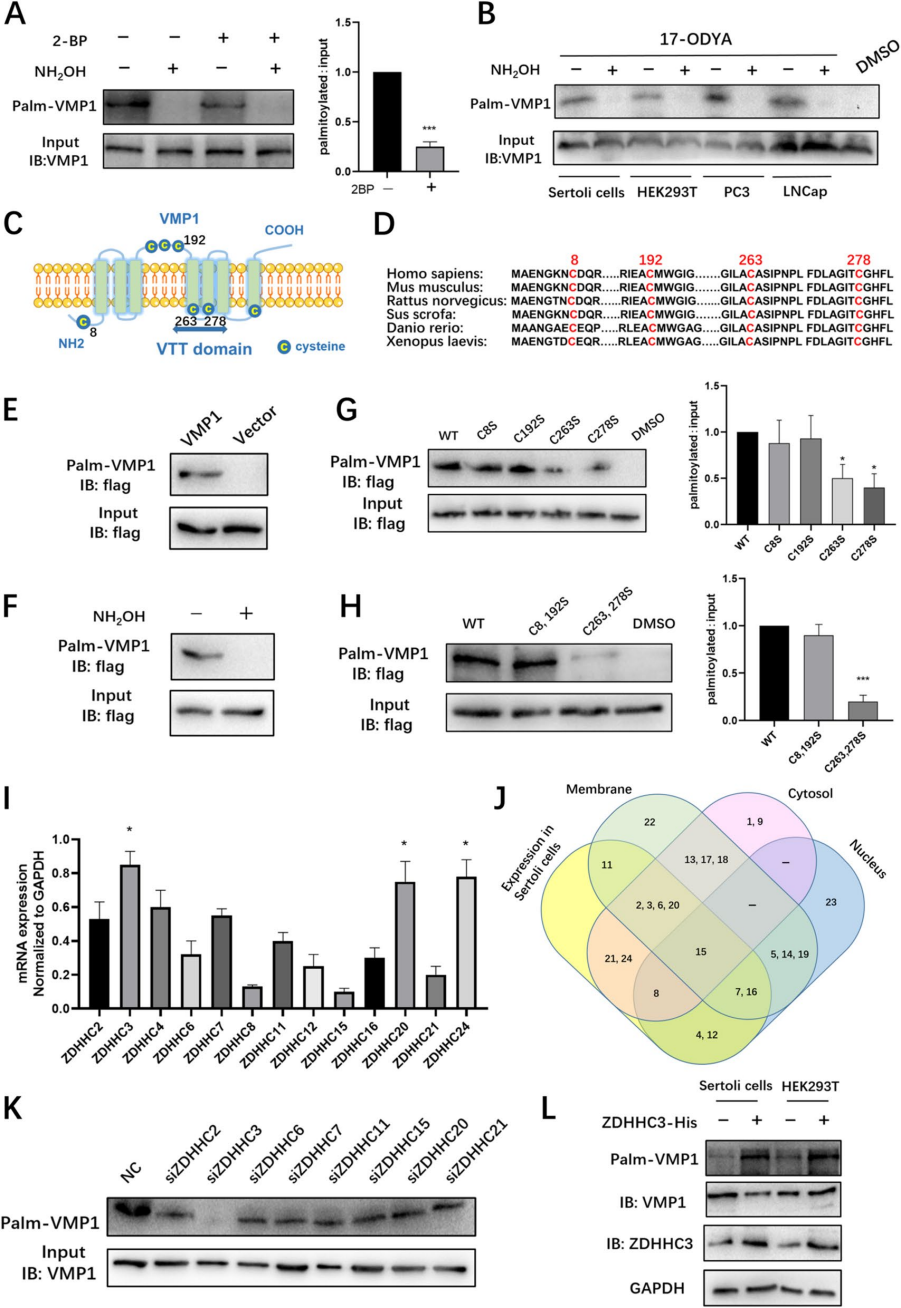
VMP1 is palmitoylated at cysteine 263 and 278 by ZDHHC3. **A** Human Sertoli cells (SCs) were metabolically labeled with 17-ODYA and then reacted with biotin-azide using click chemistry. Immunoprecipitation of biotinylated (palmitoylated) proteins from SCs showed VMP1 was palmitoylated and palmitoylation was suppressed by palmitoylation inhibitor 2-bromopalmitate (2-BP, 50 μM). Right graph: Quantified data of ratios of palmitoylated VMP1 to total VMP1 protein levels in cells treated with 2BP or control medium. ***P < 0.001. **B** Palmitoylation assay of VMP1 in SCs, HEK293T, PC3, LNCap cells that were either treated with DMSO (control) or 100 μM 17-ODYA. **C** Schematic diagram of VMP1 showed its potential palmitoylation sites and special domain (VTT domain). **D** Sequence alignments of VMP1 potential cysteine residues across different species. **E** SCs transfected with flag-tagged VMP1 or empty vector was tested with palmitoylation assay. **F** SCs transfected with flag-tagged VMP1 was tested with palmitoylation assay when NH_2_OH or Tris were added to prove palmitoylation occurred exclusively on cysteine residues. **G** VMP1 and cysteine mutant palmitoylation assays. SCs transfected with wild type flag-tagged VMP1 and the indicated single cysteine mutants were assayed for VMP1 palmitoylation levels with click chemistry. *P < 0.05. **H** SCs transfected with wild-type flag-tagged VMP1 or double cysteine site mutants were assayed for VMP1 palmitoylation level with click chemistry. ***P < 0.001. **I** Real-Time qPCR detected the mRNA of zDHHC2, 3, 4, 6, 7, 8, 11, 12, 15, 16, 20, 21, 24, gene expressions were normalized to GAPDH. *P < 0.05. **J** Venn diagram showing the expression patterns and subcellular distribution of different ZDHHCs in SCs. **K** SCs treated with indicated siRNAs targeting potential ZDHHCs (ZDHHC2, 3, 6, 7, 11, 15, 20, 21) for 72 h were assayed for VMP1 palmitoylation levels. **L** VMP1 palmitoylation was detected in HEK293T and SCs transfected with His-tagged ZDHHC3 vector or control vector. Lysate GAPDH served as a loading control

Previous palmitoyl-proteomics had proposed that VMP1 was putatively palmtoylated in different types of human cell lines [15, 28, 36]. To directly assess whether VMP1 is a palmitoylated protein, a recently developed palmitoylation assay was performed [34]. Human Sertoli cells (SCs) were metabolically labeled with 17-ODYA and then reacted with biotin-azide using click chemistry. Immunoprecipitation of biotinylated (palmitoylated) proteins from SCs showed that VMP1 was indeed palmitoylated (Fig. 1A). VMP1 palmitoylation was completely abolished by NH_2_OH treatment, indicating that it occurs exclusively on cysteine residues. Moreover, when SCs were treated with 2-BP, an irreversible palmitoylation inhibitor that abolished palmitoyl acyltransferases activities [37], VMP1 palmitoylation level was reduced evidently (Fig. 1A). Similar results were observed in different cell lines (Fig. 1B). Endogenous VMP1 was also robustly palmitoylated in 17-ODYA labeled but not in DMSO-treated samples.

In order to identify palmitoylated cysteine residues of VMP1, GPS-Palm prediction algorithm [38] was used to predict where VMP1 was palmitoylated. VMP1 had seven cysteine residues and was strongly predicted to be palmitoylated at the Cys8, Cys192 and Cys263 amino acid residues (Supplementary Fig. S1A). TMHMM-2.0 software predicted there were six transmembrane domains in VMP1. Among them, there was one conserved and important domain, named VTT domain (short for VMP1, TMEM41 and TMEM64, also known as SNARE associated domain) [39] (Fig. 1C). Sequence alignments of the Cys across different species indicated that Cys 8, 192, 263, 278 are evolutionarily conserved (Fig. 1D). To determine the exact palmitoylation sites, the cysteine-to-serine mutants (C8S, C192S, C263S and C278S) of the four potential cysteine residues were constructed and tagged with flag, the DNA sequences of the four mutants were verified by Sanger Sequencing (Supplementary Fig. S1B).

The flag-tagged wild-type VMP1 vector (WT-VMP1) was also generated, and found to be palmitoylated while flag vector was not (Fig. 1E). The palmitoylation band is gone after NH_2_OH cleaved the Cys (Fig. 1F). Interestingly, the C8S, C192S mutants showed similar palmitoylation level to WT-VMP1, indicating C8S, C192S were not palmitoylation sites. In contrast, reduced palmitoylation was found in C263S and C278S mutants but neither of them completely abolished VMP1 palmitoylation (Fig. 1G). Plasmids of multiple mutagenesis were then generated and serine replacements at both Cys263 and 278 almost completely abolished VMP1 palmitoylation, while Cys8,192 mutant displayed the same level as WT-VMP1 (Fig. 1H), suggesting Cys263 and Cys278 were the major palmitoylation sites. C263, 278S mutant was thus named as MT-VMP1.

Protein palmitoylation is mediated by a family of palmitoyl acyltransferases containing a conserved zinc-finger domain and DHHC motif (ZDHHC). To identify the predominant palmitoyl acyltransferase for VMP1 in SCs, all ZDHHCs were evaluated and 13 out of 23 ZDHHCs were found to be expressed in SCs based on the Human Protein Atlas (Supplementary Fig. S1C). Subsequently, quantitative RT-PCR of each selected gene was performed to verify the expression in SCs (Fig. 1I). Next, nuclear-localized ZDHHC 4, 12 were excluded by subcellular localization screening, while member-localized ZDHHCs were then selected (Fig. 1J). Specific siRNAs were used to respectively silence the potential candidates (including *ZDHHC2, ZDHHC3, ZDHHC6, ZDHHC7, ZDHHC11, ZDHHC15, ZDHHC20* and *ZDHHC21*). The knockdown efficacy of each siRNA was verified by RT-qPCR (Supplementary Fig. S1D). Interestingly, knockdown of ZDHHC3 greatly reduced VMP1 palmitoylation (Fig. 1K). Meanwhile, overexpression of ZDHHC3 in different cells (SCs and HEK293T) upregulated VMP1 palmitoylation distinctly (Fig. 1L). Taken together, the data demonstrate that ZDHHC3 was the major palmitoyl acyltransferase that catalyzed VMP1 palmitoylation.

### Palmitoylation affects VMP1 subcellular localization

**Fig. 2:**
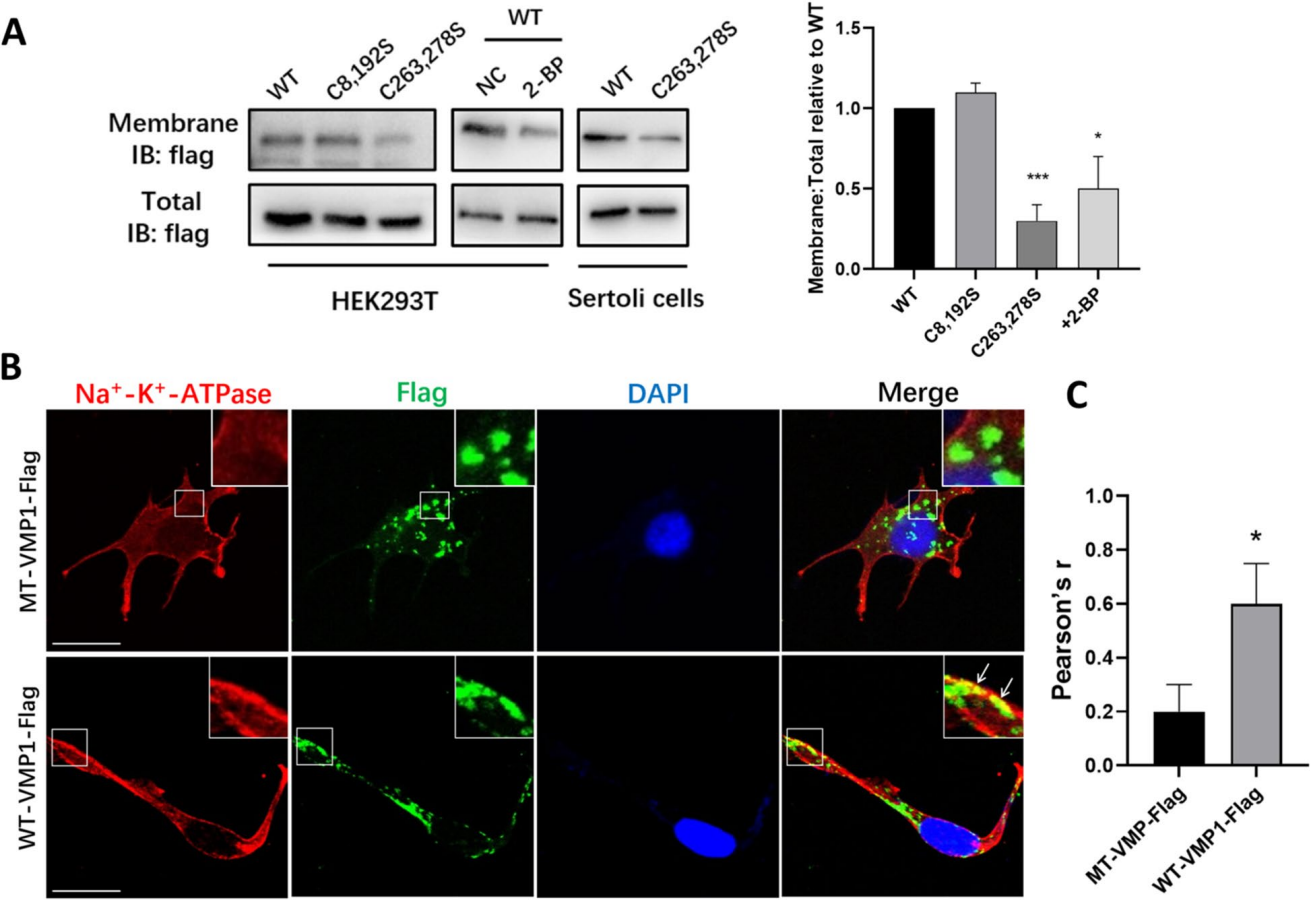
Palmitoylation affects VMP1 subcellular localization. **A** Analysis of surface expression by biotinylation. HEK293T cells expressing WT-VMP1, MT-VMP1(C8, 192S), or (C263, 278S) treated with ethanol (NC) or 2-BP, and Sertoli cells expressing WT-VMP1 or MT-VMP1 were used for surface biotinylation assays. Total and surface proteins were analyzed by western blot using anti-flag antibody. **B** Sertoli cells overexpressing WT-VMP1 or MT-VMP1 were double-immunostained with anti-Flag antibody and the antibody of Na^+^/K^+^-ATPase, the marker of plasma membrane (PM). This experiment was repeated three times independently. **C** Statistical analysis of the colocalization rate between WT-VMP1/ MT-VMP1-flag and Na^+^/K^+^-ATPase in SCs. Values are means ± SEM from *n* = 3 independent experiments. *P < 0.05, the P value was determined by two-sided Student’s t-test

As a lipid modification, palmitoylation can increase protein hydrophobicity and play a significant role in regulating protein subcellular localization and membrane association [6], therefore we investigate the effects of palmitoylation on VMP1 subcellular localization. WT-VMP1 and C8,192S demonstrated normal surface expression, whereas MT-VMP1 (C263,278S) had markedly reduced surface expression compared to WT-VMP1 in SCs and HEK293T cells (Fig. 2A). A reduction of surface distributed VMP1 was also observed in HEK293T cells treated with 2-BP (Fig. 2A).

To further verify its subcellular localization, MT-VMP1 or WT-VMP1 was overexpressed in SCs and double immunostaining was performed with plasma membrane (PM) marker, Na^+^/K^+^-ATPase antibody, to analyze VMP1 relationship with PM. The co-localization analysis revealed WT-VMP1 was primarily localized in cytoplasm and PM, however, MT-VMP1 failed to reach the PM and was mainly trapped inside the cells (Fig. 2B, C), suggesting that palmitoylation improved VMP1 membrane association and VMP1 trafficking to PM.

### VMP1 palmitoylation regulates small EV secretion in sertoli cells

**Fig. 3:**
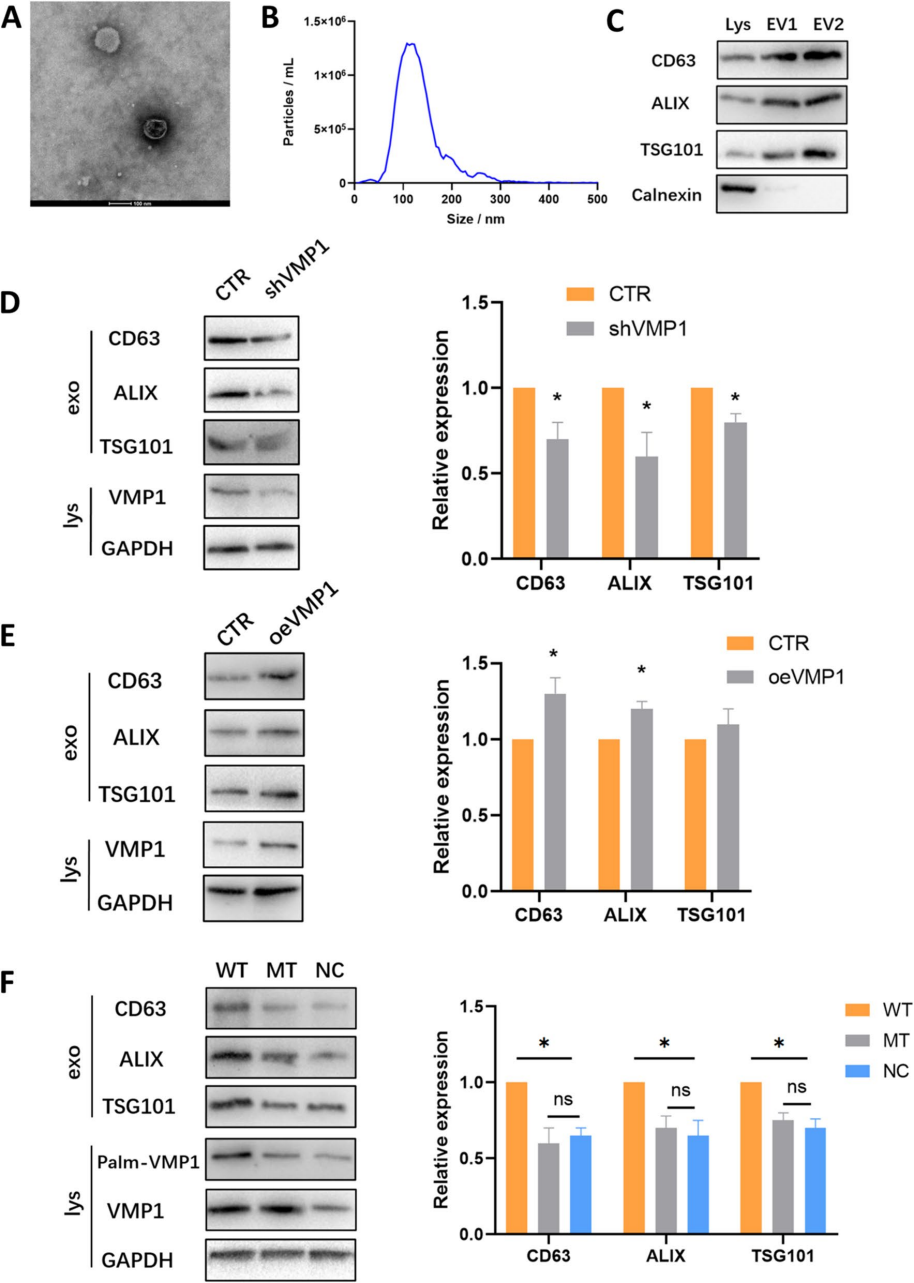
VMP1 palmitoylation regulates exosome secretion in Sertoli cells. **A** SC-derived small EVs were observed under transmission electron microscopy. **B** Representative Nanoparticle Tracking Analysis (NTA) showed the size distribution and particle number of these vesicles derived from SCs. **C** Western blot showed the expression of exosomal marker proteins (CD63, ALIX and TSG101) from two different SC-derived small EV samples, while the endoplasmic reticulum marker Calnexin of them were both negative. **D** VMP1 knockdown cells were constructed and named shVMP1. Western blot detected the expressions of exosomal protein CD63, ALIX and TSG101 in the EVs obtained from shVMP1 and CTR SC groups. **E** VMP1 overexpression cells were constructed and named oeVMP1 group. Western blot showed the expressions of exosomal protein CD63, ALIX and TSG101 in the EVs obtained from oeVMP1 and CTR SC groups. **F** Western blot detected the protein expressions of exosomal marker CD63, ALIX and TSG101 in EVs obtained from SCs transfected with WT-VMP1-Flag, MT-VMP1-Flag and empty flag vector (NC). Quantification of all exosomal protein levels in the EVs was normalized by lysate GAPDH. Each experiment was performed independently three times. Data were presented as mean ± SEM, *P < 0.05. ns: not significant

SC derived sEVs were obtained by ultracentrifugation and then observed under transmission electron microscopy (Fig. 3A). Nanoparticle Tracking Analysis (NTA) showed that the diameter of these vesicles was mainly distributed between 50-150 nm (Fig. 3B). Western blot detected the expression of exosomal marker proteins (CD63, ALIX and TSG101) from two different SC-derived sEV samples, while the endoplasmic reticulum marker Calnexin were both negative (Fig. 3C).

To explore the role of VMP1 in sEV secretion, cell lines with VMP1 knockdown and overexpression (shVMP1 and oeVMP1 group) were constructed (Fig. 3D, E). NTA measurement showed that the sEV amount in shVMP1 group was significantly less than that in the control group (CTR group) (Supplementary Fig. S2A), with no effect on particle size. The expressions of CD63, ALIX and TSG101 were much lower in shVMP1 group (Fig. 3D). Meanwhile, oeVMP1 group secreted more sEVs compared with CTR group (Supplementary Fig. S2B), and expressions of CD63, ALIX were also significantly higher in the oeVMP1 group (Fig. 3E). Altogether, these results suggested that VMP1 regulated sEV secretion in SCs.

Interestingly, administration of palmitoylation inhibitor 2BP to oeVMP1 group greatly decreased the sEV release amount to the level that resembled the CTR group (Supplementary Fig. S2C). This indicated palmitoylation played a key role in the regulation of sEV release by VMP1. To further explore the effect of VMP1 palmitoylation on sEV secretion in SCs, WT-VMP1 (WT group), MT-VMP1 (MT group) and negative control (NC group) vector were transfected into SCs. NTA measurement showed the sEV release amount in WT group was much more than that in MT group and NC group (Supplementary Fig. S2D). The expressions of CD63, ALIX and TSG101 in sEVs from WT group were also significantly higher than those of the other two groups (Fig. 3F), while there was no statistical difference between MT and NC group. These results demonstrated that inhibition of VMP1 palmitoylation reduced sEV secretion in SCs, indicating palmitoylation of VMP1 could regulate sEV secretion in SCs.

### Inhibition of VMP1 palmitoylation influences endosome-MVB-lysosome system

**Fig. 4:**
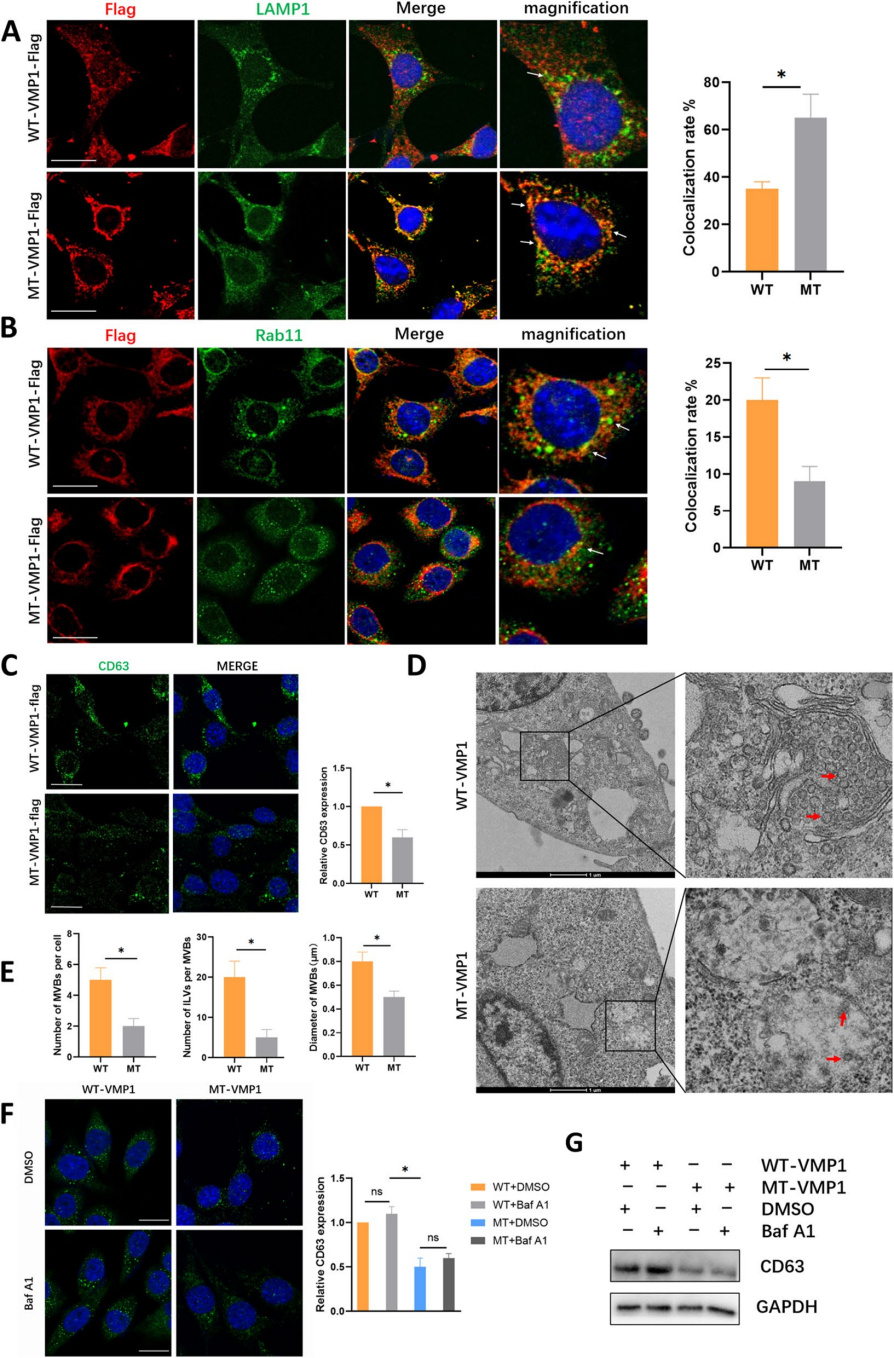
Inhibition of VMP1 palmitoylation influences endosome-MVB-lysosome system. **A** Representative immunofluorescence images for the colocalization between ectopically expressed WT-VMP1/ MT-VMP1-Flag and LAMP1, the marker for lysosome. The white arrows in the magnified photos indicate colocalized VMP1 and LAMP1 in SCs. Right panel: Statistical analysis of the co-localization rate between Flag and LAMP1 in two groups. **B** Representative immunofluorescence showing the colocalization between WT-VMP1/ MT-VMP1-Flag and Rab11b, the specific marker for recycling endosome. Right panel is the co-localization analysis of Flag and Rab11b in two groups. **C** Representative immunofluorescence shows CD63 expression (marker of MVBs) in SCs transfected with WT-VMP1 or MT-VMP1-Flag. Right graph: quantification of relative CD63 fluorescence intensity of MT group normalized to WT group (control). **D** Electron microscopy displays the morphology and size of MVBs in SC transfected with MT-VMP1 or WT-VMP1-Flag (The red arrow represents the intraluminal vesicles in MVBs). **E** The statistical analysis of MVB size and number and intraluminal vesicles per MVB between two groups in image D. **F** Immunofluorescence of CD63 expression (MVB marker) in SCs overexpressing WT-VMP1 or MT-VMP1 treated with Baf A1 (20 nM) or DMSO. Right graph: quantification of the relative CD63 fluorescence intensity in the four groups. Data were presented as mean ± SEM, *P < 0.05. ns: not significant. **G** Western blot of CD63 expression (MVB marker) in SCs transfected with WT-VMP1 or MT-VMP1 treated with Baf1 or DMSO. Representative results from three independent experiments are shown

To reveal the mechanism by which VMP1 palmitoylation regulated sEV secretion, endosome-MVB-lysosome system was studied. We first investigated the localization of mutant VMP1 and wild-type VMP1 on endosomes and lysosomes. Interestingly, disruption of VMP1 palmitoylation by mutation (MT-VMP1) reduced the co-localization of VMP1 on Rab7b-labelled late endosomes (Supplementary Fig. S3B), while had no significant influence on the co-localization of VMP1 and EEA1-labelled early endosomes (Supplementary Fig. S3A). Additionally, MT-VMP1 substantially increased the colocalization of VMP1 with lysosomes (marked by LAMP1), while causing substantial decrease on Rab11-marked recycling endosomes (Fig. 4A, B), indicating that inhibition of VMP1 palmitoylation affected VMP1 recycling efficiency and increased its recruitment to lysosome for degradation.

To further investigate the mechanisms, MVBs were studied. The Immunofluorescence showed that the expression levels of MVB marker proteins CD63 and HRS (an ESCRT-0 Subunit) were significantly reduced by MT-VMP1 transfected SCs (Fig. 4C, Supplementary Fig. S3C), suggesting that loss of VMP1 palmitoylation could reduce MVBs in SCs. Moreover, palmitoylated VMP1 induced a more perinuclear and clustered localization of CD63, expressed in a granular form and larger spot. Similarly, electron microscopy showed the size of MVBs was smaller, and the numbers and density of MVBs and ILVs (represented by the red arrow) per MVB were also decreased when VMP1 palmitoylation was inhibited (Fig. 4D, E). This indicated that VMP1 palmitoylation may affect the quantity, size and distribution of MVBs, and thus regulate sEV secretion in SCs.

To further test whether inhibition of VMP1 palmitoylation suppressed MVB formation or promoted MVB degradation, Bafilomycin A1 (Baf A1) was used to inhibit lysosome H^+^-ATPase activity and degradation ability in SCs. Interestingly, CD63 expression, representative of MVBs, was not significantly changed after administration of Baf A1 (Fig. 4F), which revealed that inhibition of lysosomal activity may not lead to reduction of MVB degradation in either MT-VMP1 or WT-VMP1 group. Similar results were observed when western blot of CD63 was conducted (Fig. 4G). The data confirmed that lysosome degradation was not the major factor that caused the reduced MVBs in palmitoylated site-mutant VMP1, and further suggested that inhibited biogenesis of MVBs may take the major responsibility for the decreased secretion of sEVs when VMP1 palmitoylation was suppressed.

### Inhibition of VMP1 palmitoylation abolishes the interaction between VMP1 and ALIX

**Fig. 5:**
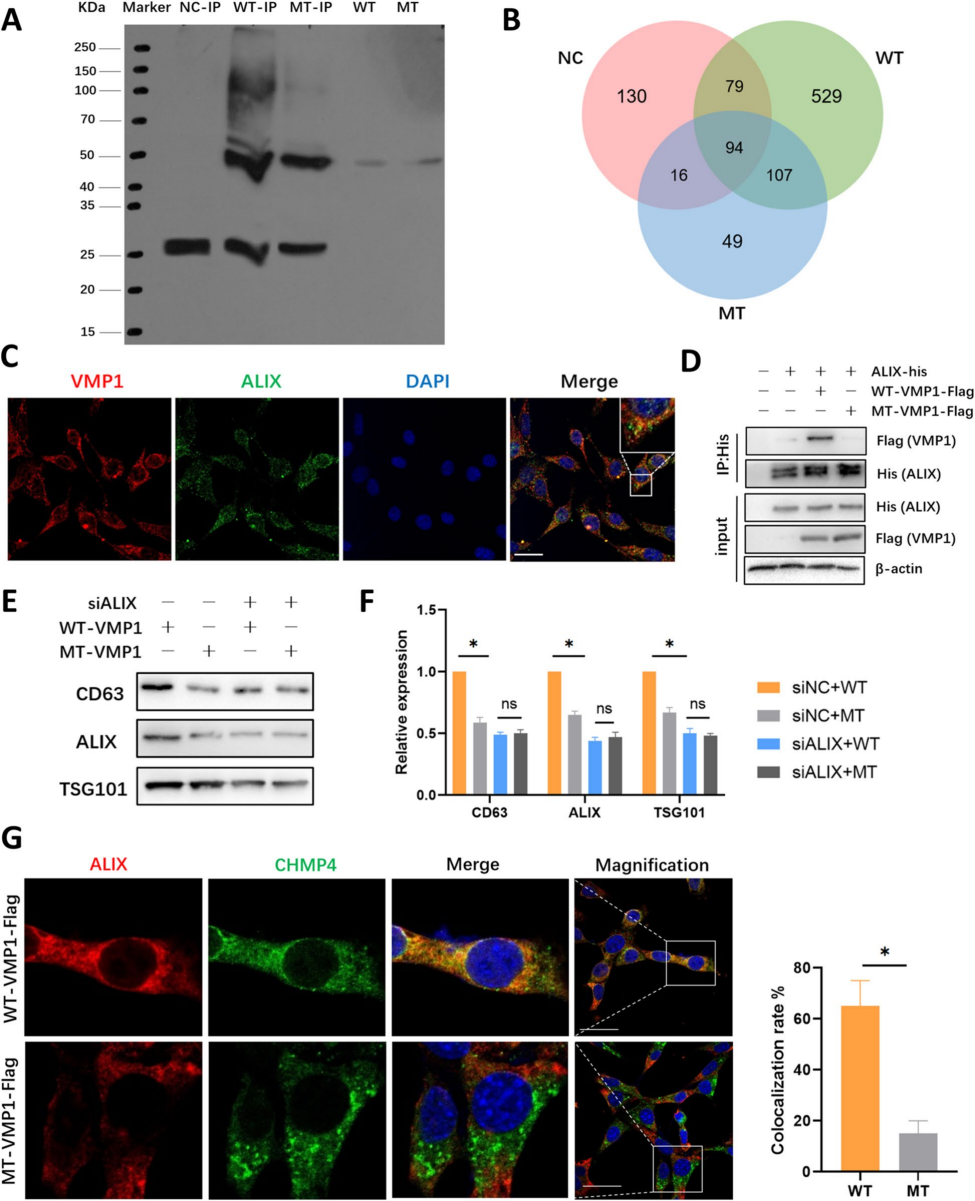
Inhibition of VMP1 palmitoylation abolished the interaction between VMP1 and ALIX. **A** SCs were transfected with WT-VMP1-flag, MT-VMP1-flag or flag-tagged control plasmid (NC), and then conducted immunoprecipitation with anti-Flag. Total lysates and flag immunoprecipitates were then blotted. **B** Venn diagram of mass spectrometry results showed proteins that interacted with Flag tagged WT-VMP1, MT-VMP1, NC vectors. **C** Double-immunofluorescence of endogenous VMP1 and ALIX expression in SCs. The co-localization area is magnified in white box. **D** SCs were transfected with His-ALIX and WT-VMP1 or MT-VMP1, and tested for the interaction of exogenous VMP1 and ALIX by immunoprecipitation with anti-His antibody. β-actin served as a loading control. **E** The western blot of exosomal marker protein CD63, ALIX, TSG101 in sEVs obtained from SCs transfected with WT-VMP1 or MT-VMP1 and siALIX or siNC vector. **F** Quantification of relative exosomal protein CD63, ALIX, TSG101 in **E** of three independent experiments. Data were presented as mean ± SEM, *P < 0.05. ns: not significant. **G** Double immunofluorescence of CHMP4 and ALIX expression in SCs transfected with WT-VMP1 or MT-VMP1 vector. Right panel: Statistical analysis of the co-localization rate between ALIX and CHMP4 in the two groups. The experiment was repeated three times

To gain further insight into the mechanisms by which VMP1 palmitoylation affects MVB biogenesis and sEV secretion in SCs, WT-VMP1, MT-VMP1 and control vectors tagged with Flag were transfected into SCs respectively, followed by immunoprecipitation with flag (Fig. 5A) and mass spectrometry of the immunoprecipitates. Venn diagram of protein interactome showed that proteins interacting with WT-VMP1, MT-VMP1, NC groups were both overlapped and different (Fig. 5B). GO and KEGG analyses were performed for WT-VMP1, MT-VMP1 and NC interacted proteins (Supplementary Fig. S4A, B). Proteins that interacted with WT-VMP1 were enriched in pathways like amino acid and glycolipid metabolism, transportation, protein sorting and degradation, whereas the interactions of MT-VMP1 were slightly different, suggesting that inhibition of palmitoylation may affect VMP1 function through regulating the interactions with VMP1 and its downstream effectors. Subsequently, the proteins present in WT-VMP1 but excluded in MT-VMP1 or NC groups were further selected and analyzed (Supplementary Fig. S4C). Among those proteins, ALIX (encoded by PDCD6IP gene) is recognized as a bona fide regulator and marker of exosomes. ALIX plays an important role in MVB biogenesis and EV secretion through regulating cargo protein sorting and ILV formation [40, 41]. As expected, ALIX was co-localized with endogenous VMP1 (Fig. 5C). Further verification by Co-IP in HEK293T showed that Alix-His interacted with WT-VMP1 rather than MT-VMP1 (Fig. 5D), confirming that disruption of VMP1 palmitoylation affected the interaction between VMP1 and ALIX.

In order to probe whether abolished interaction between VMP1 and ALIX may affect ALIX function and sEV secretion, ALIX knockdown model of SCs by siALIX was constructed (Supplementary Fig. S4D). SCs expressing siALIX or the negative control vector (siNC) were respectively transfected with WT-VMP1 or MT-VMP1, divided into four groups. As expected, the amount of sEV secretion in two siALIX group were distinctly reduced (Fig. 5E). In siNC groups, the expression of CD63, ALIX and TSG101 in MT-VMP1 was significantly decreased when compared with WT-VMP1. However, after ALIX deletion, there was no significant difference between WT-VMP1 and MT-VMP1 group (Fig. 5E, F). This indicated that ALIX played an indispensable role in the regulation of VMP1 palmitoylation-mediated sEV secretion process. It is well-known that ALIX regulates ILV formation through interacting with CHMP4 in ESRCT III [40]. The co-localization of ALIX and CHMP4 in MT-VMP1 group was significantly weakened when compared to the SCs expressing WT-VMP1 (Fig. 5G). Taken together, the disruption of VMP1 palmitoylation may affect the recruitment of ESCRT III and formation of MVBs via VMP1-ALIX-CHMP4 axis, leading to reduced sEV release.

### VMP1 palmitoylation affects SSCLC self-renewal in SC-SSCLC co-culture system

**Fig. 6:**
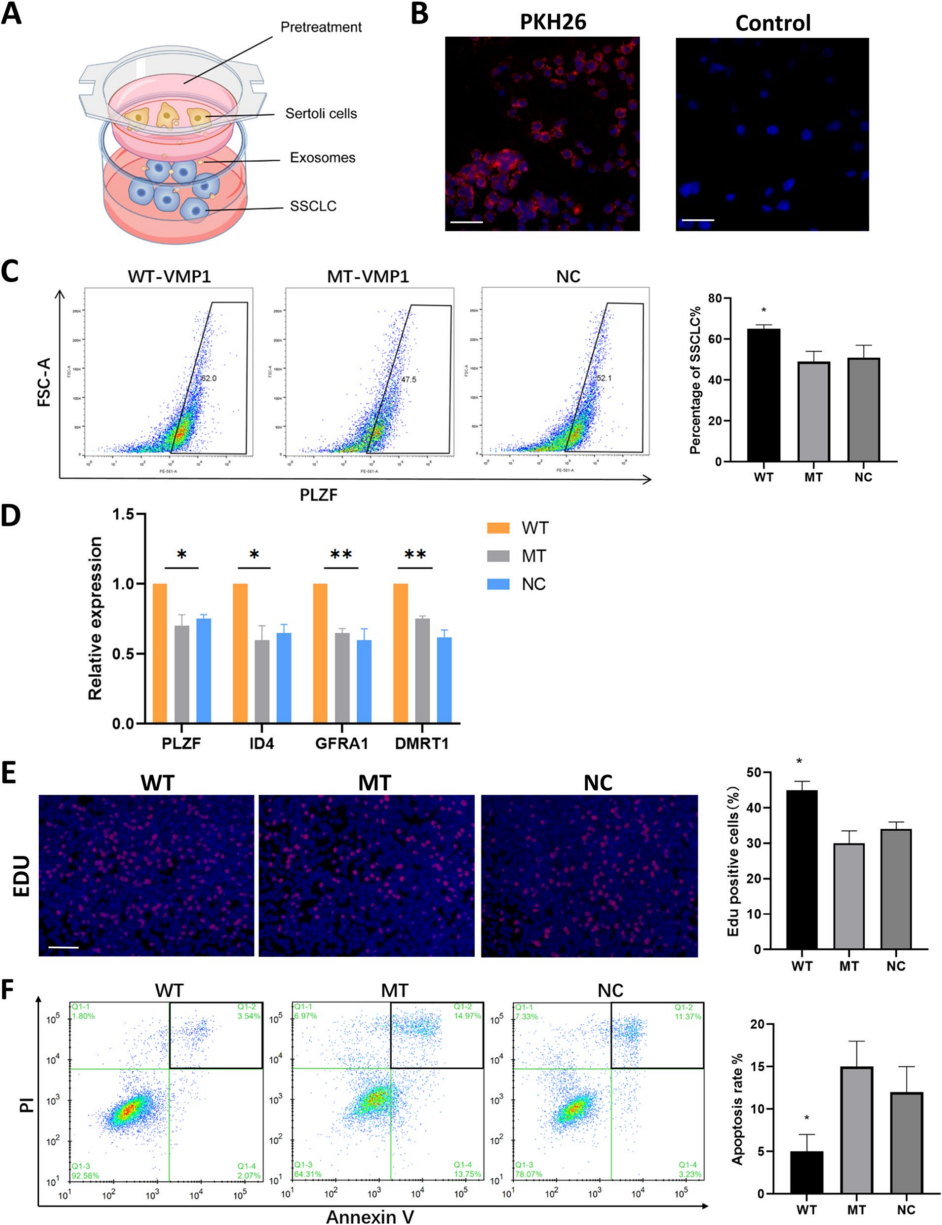
VMP1 palmitoylation affects SSCLC self-maintenance and proliferation in co-culture. **A** Schematic diagram of co-culture of SSCLCs with SCs transfected by WT-VMP1-Flag, MT-VMP1-Flag or NC plasmid, with SSCLCs in the lower chamber and SCs in the upper chamber of a Transwell (0.4 μm). **B** SCs-derived sEVs were labeled by PKH26 dye and then incubated with SSCLCs for 24 h. Representative fluorescence images of PKH26-labelled (red) SSCLCs. The control group was treated with the medium of dye solvent without PKH26. **C** SCs were transfected with WT-VMP1-Flag, MT-VMP1-Flag or NC plasmid respectively in the upper layer. After 24 h of co-incubation, the proportion of PLZF positive cells in the lower layer was assessed by flow cytometry to represent SSCLC ratio in WT-VMP1, MT-VMP1, NC groups on the 12th day of induction. **D** Real time qPCR experiment showed the expression of SSC-related genes PLZF, GFRα1, ID4 and spermatogonial related genes DMRT1 in WT-VMP1, MT-VMP1 and NC groups. **E** The proliferation rate of SSCLCs was tested by EDU assay in WT-VMP1, MT-VMP1 and NC groups. **F** The apoptosis rate of SSCLCs was detected by FITC-Annexin V flow cytometry in WT-VMP1, MT-VMP1 and NC groups. Black boxes represent the apoptosis ratio of SSCLCs. Three independent times of each experiment were performed and statistically analyzed. Data were presented as mean ± SEM, *P < 0.05

Human SSCs are difficult to isolate and culture, while induced differentiation of spermatogonial stem cell-like cells (SSCLCs) from human iPSC possess the biological function of SSCs and the potential to further differentiate into haploid [42]. A co-culture system of SCs and SSCLCs was established, with SSCLCs in the lower layer and sEVs transported from the transwell (Fig. 6A). SCs-derived sEVs were labeled by PKH26 and incubated with SSCLCs for 24 h. Red fluorescence staining was observed in SSCLCs while control group was not (Fig. 6B), suggesting that sEVs derived from SCs could be taken in by SSCLCs, which had also been testified by other researchers previously [31, 32].

Since VMP1 palmitoylation could affect sEV secretion in SCs, whether VMP1 palmitoylation had an influence on SC-SSCLC co-culture and SSC maintenance was further explored. Three shVMP1 SC groups were respectively transfected with WT-VMP1-Flag, MT-VMP1-Flag and NC plasmid. After 24 h co-culture, no significant difference was found in the density of SCs and SSCLC morphology between the three groups. The proportion of PLZF positive cells were applied to represent SSCLC ratio on the 12th day of induction [43]. It turned out the proportion of PLZF positive cells in WT-VMP1 group were significantly higher than the other groups, while there was no significant difference between MT-VMP1 and NC groups (Fig. 6C). The data indicated VMP1 palmitoylation in SCs played an important role in the regulation of SSCLC self-renewal.

RT-qPCR results showed that on day 12 of SSCLC induction, the expression of SSC marker genes PLZF, GFRα1, ID4 and spermatogonial related genes DMRT1 in MT-VMP1 and NC groups were substantially lower than WT-VMP1 group (*P* < 0.05) (Fig. 6D). Moreover, MT-VMP1 overexpression in SCs induced significantly decreased cell proliferation and increased apoptosis rate of SSCLCs in co-culture system (Fig. 6E, F). Taken together, the data demonstrated that VMP1 palmitoylation in SCs may affect SSCLC maintenance, proliferation and apoptosis in vitro.

### Palmitoylation inhibitor 2BP affects sEV secretion in Sertoli cells and causes apoptosis of seminiferous tubules in mice

**Fig. 7:**
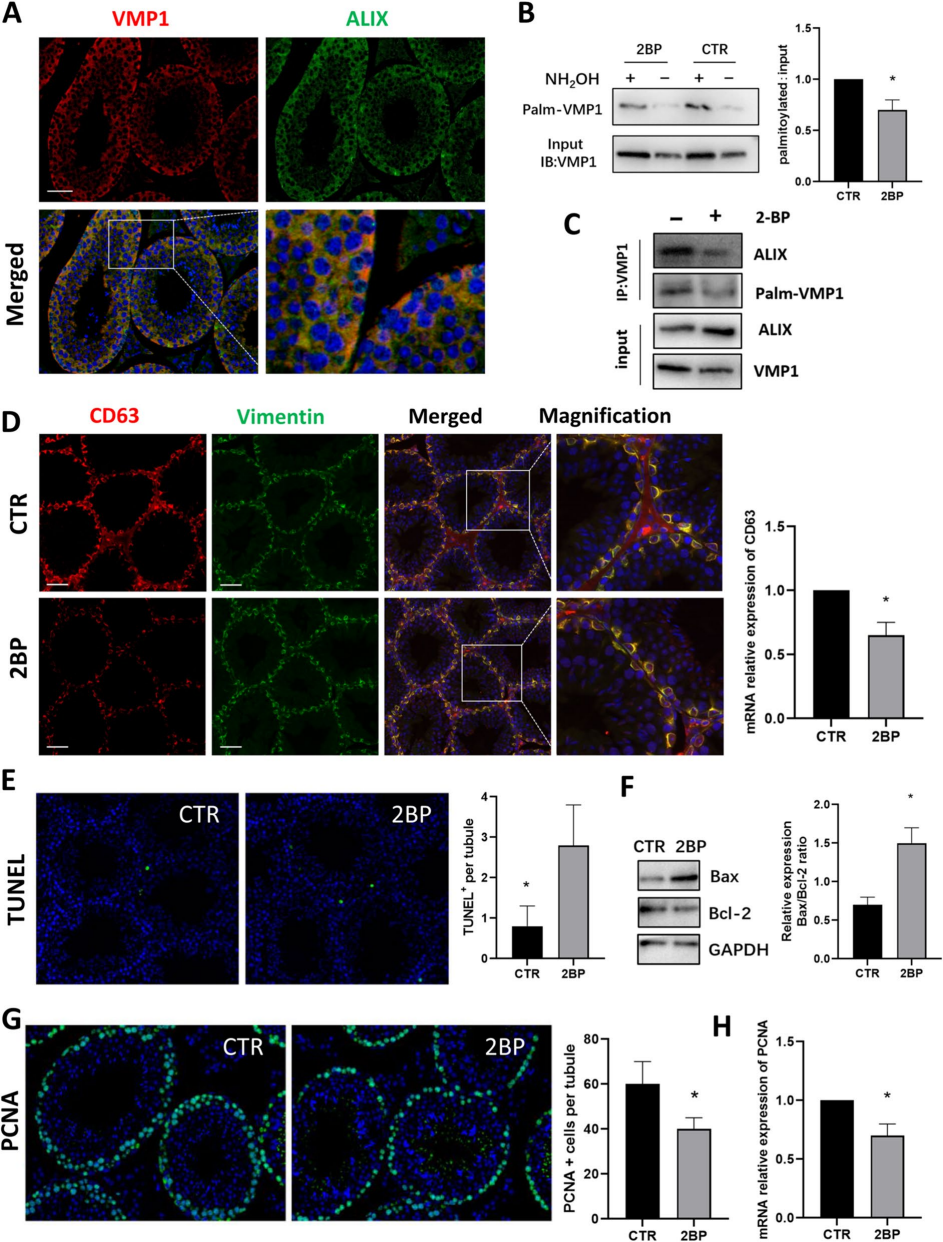
palmitoylation inhibitor 2BP affects sEV secretion in SCs and causes apoptosis of seminiferous tubules in mice. **A** Double immunofluorescence staining showed that VMP1 was colocalized with ALIX in normal adult mouse testis. **B** Immunoprecipitation and Acyl-Biotin Exchange assay of VMP1 palmitoylation in control mouse groups (CTR) and 2BP mouse groups (2BP) that were intraperitoneally injected with palmitoylation inhibitor 2BP for 3 months. Right graph: Quantified data of ratios of palmitoylated VMP1 to total VMP1 protein levels in CTR or 2BP groups. **C** Co-immunoprecipitation of VMP1 interaction with ALIX in CTR (left) and 2BP (right) mouse testes. **D** Confocal images of double immunofluorescence staining of SC marker Vimentin and CD63 (the marker of exosomes and MVBs) in CTR or 2BP treated mouse testes. CD63 (red) and Vimentin (green) immunoreactivity are merged and magnified. Right panel is the quantification of CD63 fluorescence intensity. **E** TUNEL staining of seminiferous tubules in CTR or 2BP testes. The positive cells (green) are representative of apoptosis. Right graph: quantification of the number of positive cells per tubule in two groups. **F** Western blot of apoptosis-related protein Bax and Bcl-2 expression in CTR or 2BP testes. GAPDH served as a loading control. Right panel is quantification of the Bax/Bcl-2 expression ratio in CTR or 2BP. **F** Immunofluorescence staining PCNA (proliferating cell nuclear antigen), a marker for cell proliferative activity, in CTR or 2BP testes. Quantification of PCNA^+^ cells per seminiferous tubules was carried out in testes of two groups. **H** Quantitative PCR assay of relative mRNA levels of PCNA expression in CTR or 2BP testes. All scale bars = 50 μm, Data were obtained from three to five independent experiments and were presented as mean ± SEM, *P < 0.05

To explore VMP1 expression in vivo, immunofluorescence was performed on 1-week old mouse testes and displayed VMP1 was expressed in both SCs and spermatogenic cells (Supplementary Fig. S5A). Meanwhile, in adult mice (6-week old), VMP1 was mainly expressed in SCs, colocalized with SC marker Vimentin (Supplementary Fig. S5B). Moreover, double immunostaining staining showed that VMP1 was colocalized with ALIX (Fig. 7A), suggesting VMP1 may interact with ALIX in vivo.

Due to technical barrier, genetic mouse model with VMP1 palmitoylation site mutant in SCs had not been established yet. In order to get a preliminary understanding of VMP1 palmitoylation and the role palmitoylation played in spermatogenesis, adult mice were intraperitoneally injected with palmitoylation inhibitor 2BP for three months to simulate the effect of VMP1 palmitoylation deficiency in vivo. As expected, palmitoylation of VMP1 was detected in testis by immunoprecipitation and Acyl-Biotin Exchange assay [35], and the level of VMP1 palmitoylation was evidently decreased after 2BP injection (Fig. 7B). CO-IP showed the interaction of VMP1 and ALIX declined greatly when palmitoylation was inhibited by 2BP treatment (Fig. 7C), which is consistent with the results of in vitro experiments. Moreover, CD63, the marker for exosomes and MVBs, was significantly lower in 2BP group when compared with the control group (CTR group), demonstrating sEV secretion process was blocked by palmitoylation inhibitor in SCs (Fig. 7D). TUNEL staining of seminiferous tubules showed the apoptosis rate of the 2BP group was significantly higher than that of the CTR group (Fig. 7E). Administration of 2BP to mice also significantly elevated Bax level and reduced Bcl-2 expression in testes, and the increased ratio of Bax/Bcl-2 was representative of cell apoptosis (Fig. 7F). PCNA (proliferating cell nuclear antigen), a marker for cell proliferative activity, was expressed at a lower level in 2BP group compared to control (Fig. 7G, H). Collectively, the results indicated inhibition of palmitoylation may affect sEV secretion in SCs, and it was closely associated with the apoptosis and proliferation of spermatogenic cells. The causal relationship needs to be further verified in palmitoylation site mutant mice.

## Discussion

This study provides a novel mechanism for the regulation of sEV biogenesis and secretion by VMP1 palmitoylation, which is pivotal in SC-SSC cellular communication and male fertility. In the study, we first identified the palmitoylation sites (Cys 263 and 278) of VMP1 and its regulation by ZDHHC3, and then revealed a crucial role of VMP1 palmitoylation in controlling MVB formation and exosome secretion. The underlying mechanism involved that palmitoylation affected VMP1 subcellular localization and trafficking, ILV and MVB formation. Moreover, palmitoylation was required for VMP1 interaction with ALIX and ALIX/CHMP4 mediated-sEV release. By taken SC-SSCLC communication as a model, it further demonstrated sEV secretion regulated by VMP1 palmitoylation in SCs may play a significant role in SSC niche.

Researchers found that VMP1 controls ER contacts with other organelles (like endosomes, lipid droplets, mitochondria) through interacting with SERCA (sarcoplasmic reticulum calcium ATPase) [44]. VMP1 deficiency in the midbrain dopaminergic neurons led to disrupted autophagy flux and necroptosis [45]. In our study, VMP1 was found to affect sEV secretion in SCs, we thus made the hypothesis that VMP1 may regulate MVB formation and sEV secretion through palmitoylation. Subsequently, site-directed mutagenesis was performed at four potential Cys, and palmitoylation level was significantly decreased after mutation of C263, C278 to serine, indicating these two sites were the major palmitoylation sites. Interestingly, the two palmitoylation sites happen to be localized in the VTT domain of VMP1 [46]. VTT has ion coupling transport function predicted by bioinformatics [39]. It plays an important role in autophagy, lipid transport and membrane homeostasis, etc. Mutations of the conserved glycine residues [47] in VTT domain or deletion of it [48] lead to function inactivation. Hence, inhibition of VMP1 palmitoylation by site-directed mutations may affect the membrane affinity, conformation and subcellular localization of VMP1, and then abolish the function of VMP1.

Protein post-translational modification has emerged as a novel regulator of EV biogenesis and secretion [49, 50]. A previous study identified a certain proportion of exosomal and endosomal proteins were palmitoylation targets [15]. However, whether palmitoylation can regulate EV biogenesis is largely unexplored. The secretion process of exosomes mainly includes ILVs formation, MVBs degradation with lysosomes, and MVBs fusion with plasma membrane released as exosomes. The production of ILV/MVB is in dynamic balance with the degradation of MVB in lysosome [51]. In our study, inhibition of VMP1 palmitoylation by site mutation reduced sEV secretion to a large extent. In fact, MT-VMP1 reduced the number and size of MVBs, while the number of ILVs in MVB also decreased. Moreover, the amount of MVBs did not significantly increase after the use of lysosomal inhibitors, so we speculated the reduction of MVBs was caused by the decrease of ILVs/MVBs formation, rather than the increase of lysosomal degradation. VMP1 palmitoylation controls sEV secretion in SCs mainly through regulating ILV and MVB formation.

In order to further explore the molecular mechanism, we analyzed the differential enrichment of interacting proteins with WT-VMP1 and MT-VMP1 by protein immunoprecipitation and mass spectrometry. It turned out that inhibition of VMP1 palmitoylation could reduce the interaction of VMP1 with a number of proteins enriched in vesicle transport and sEV formation pathway, such as Rab9A, Rab1B, COPII, ALIX, etc. We then focused on the verification of specific protein regulating ILVs formation, namely the ESCRT III-associated protein ALIX, and further confirmed the co-localization and interaction between VMP1 and ALIX by immunofluorescence and co-IP. In addition, when VMP1 palmitoylation sites were mutated, the interaction between VMP and ALIX was abolished. Therefore, we considered that VMP1 palmitoylation was essential for interaction with ALIX and proper function of ALX. ALIX, known as an exosomal marker, can interact with CHMP4 in ESCRT III, and it is involved in the process of cargo protein sorting and ILV formation into MVBs, the key to sEV secretion [52]. While inhibiton of ALIX can reduce sEV secretion and affect the protein content in sEVs distinctly. Moreover, it is well-known that the BRO1 domain of ALIX interacts with CHMP4 to mediate the recruitment of ESCRT III to MVBs, resulting in ILV vesicle formation [53, 54]. Knockdown of CHMP4 significantly reduce sEV secretion, and site mutation (amino acid I212D) of ALIX that prevents ALIX-CHMP4 interaction also greatly reduced ILV formation and sEV production [55]. In the study, when ALIX was knocked down, VMP1 palmitoylation no longer had a regulatory effect on sEV release, indicating this regulatory effect was mainly dependent on ALIX and the interaction. When VMP1 palmitoylation was inhibited, the interaction of ALIX and its downstream effector CHMP4 was reduced, indicating that ALIX's role in ESCRT III recruitment was impaired. Hence, ALIX may play an important role in palmitoylated VMP1-mediated regulation of MVB formation and sEV secretion.

SSCs are the stem cells that support spermatogenesis. SCs are the main cells comprised the SSC niche, which are the key to dictating SSC fate decisions and spermatogenesis. Previously, we developed an induction protocol for SSCLCs from human iPSCs with high induction efficiency (about 60–70%) [33], while the functions of SCs towards SSCs had not been tested in vitro. SC-SSCLC co-culture experiment is a representative in vitro model for intercellular communication research in spermatogenesis process. Through SSCLC co-culture with human SCs, transfer of sEVs from SCs to SSCLCs was observed, and we demonstrated that palmitoylated VMP1 in SCs enhanced SSCLCs formation and proliferation, and decreased the apoptosis of SSCLCs, whereas the mutant non-palmitoylatable form of VMP1 did not. However, there is a caveat to this part, PKH26 transfer between cells through a transwell could occur through other membranes than exosomes, including ectosomes, lipid micelles, or other non-vesicular factors. Therefore, the results cannot be over interpreted. To prove this, we improved the system by using the sEV secretion inhibitor GW4869 to show that sEVs were the major contributing factor to SSCLC growth in another paper (unpublished). In vivo, VMP1 was mainly expressed in the SCs of adult male mice. Pharmacological inhibition of VMP1 palmitoylation by 2BP distinctly decreased MVB marker CD63 expression and thus sEV secretion in SCs, which was highly correlated with the apoptosis and proliferation of spermatogenic cells in mice. Still, we need to establish VMP1 palmitoylation-site mutant mice to further testify the functions of palmitoylated VMP1 and the causal relationship between palmitoylation and cell apoptosis or proliferation in testes. Together, the co-culture and in vivo data indicated that VMP1 palmitoylation in SCs might play a role in SSC self-renewal, proliferation and apoptosis, and presented SC-derived sEVs as new components in SSC niche.

## Conclusions

**Fig. 8:**
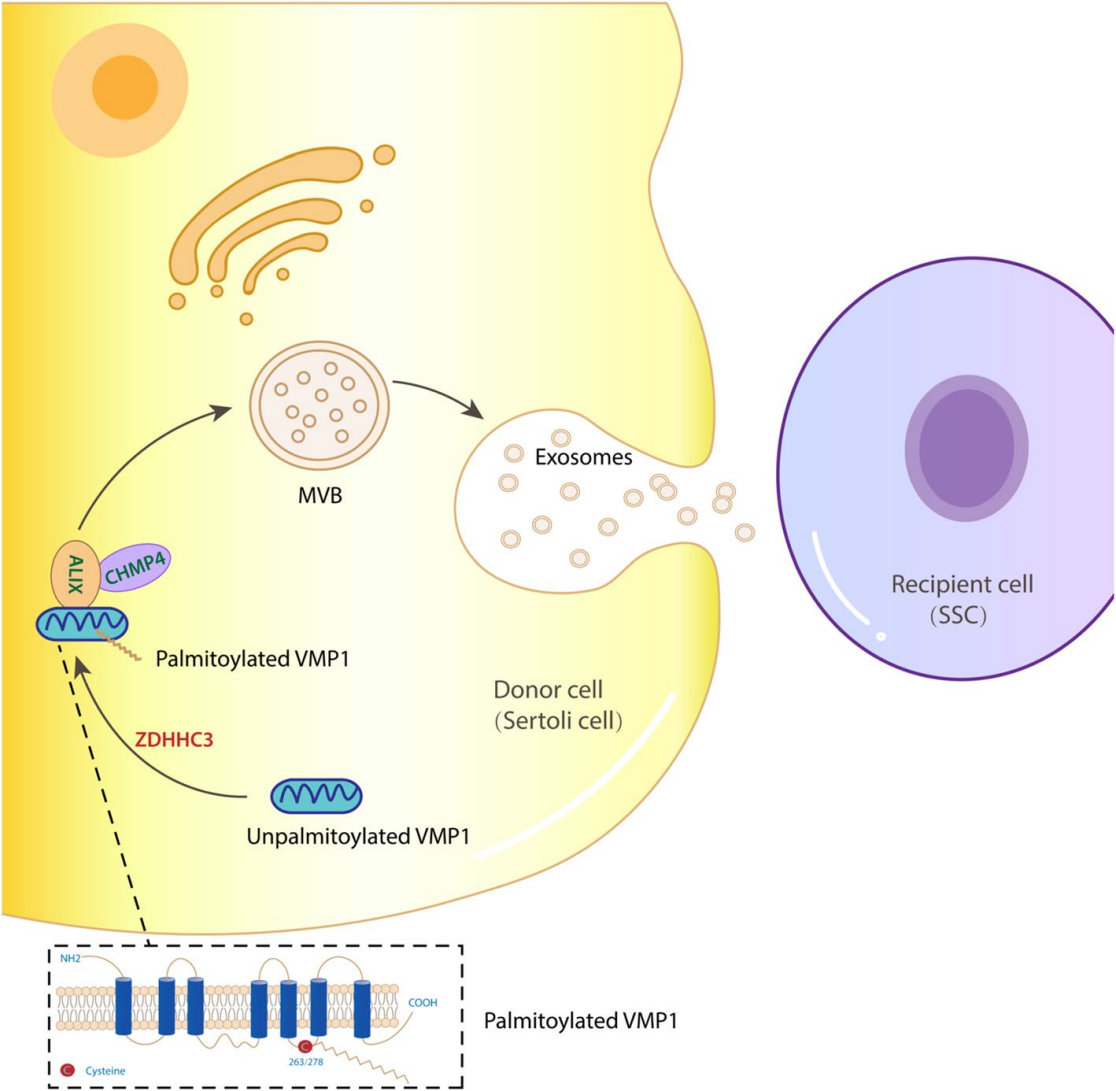
A schematic representation of a model for the role of VMP1 palmitoylation in the regulation of sEV secretion. VMP1 palmitoylation promotes the interaction between VMP1 and ALIX, thus enhances MVB formation and sEV secretion in Sertoli cells, which is essential for SSC maintenance

In summary (shown in Fig. 8), VMP1 is palmitoylated at Cys 263, 278 by ZDHHC3. Palmitoylation determines the subcellular localization of VMP1 on endosomal-lysosomal-MVB system, and also affects protein interaction between VMP1 and ALIX. In fact, VMP1 regulates MVB formation and sEV secretion in palmitoylation-dependent manner. Inhibition of VMP1 palmitoylation suppresses cellular MVB biogenesis and VMP1/ALIX/CHMP4 signaling. The novel role of palmitoylated VMP1 in sEV secretion further influences SC-SSC communication and SSC self-renewal, proliferation condition. In addition to the role in SSC niche, palmitoylated VMP1 probably holds high potential as a target in other diseases in which sEVs are involved. Given that sEVs play a pivotal role in various cell functions and signal transduction, the exact molecular mechanisms implicated in sEV secretion warrant further exploration.

### Supplementary Information

**Supplementary Material 1.**

**Supplementary Material 2.**

## Data Availability

The datasets used and/or analyzed during the current study are available from the corresponding author on reasonable request.

## Abbreviations

2-BP: 2-Bromopalmitate
17-ODYA: 17-Octadecynoic acid
ABE: Acyl-biotinyl exchange
Baf A1: Bafilomycin A1
ESCRT: Endosome sorting complexes required for transport
ILV: Intraluminal vesicle
IP: Immunoprecipitation
iPSC: Induced pluripotent stem cell
MVB: Multivesicular body
NTA: Nanoparticle Tracking Analysis
PAT: Palmitoyl acyltransferase
SSC: Spermatogonial stem cell
SC: Sertoli cell
VMP1: Vacuole membrane protein 1
VPA: Valproic acid
