# Supplementary material for: Palmitoylation of vacuole membrane protein 1 promotes small extracellular vesicle secretion via interaction with ALIX and influences intercellular communication
Source: Cell Commun Signal. 2024 Feb 26;22:150. doi: 10.1186/s12964-024-01529-6 (PMC10895845; doi:10.1186/s12964-024-01529-6)

Supplementary Figure S1-5

Supplementary Fig. S1A GPS-Palm predicted that there were seven cysteine residues in VMP1 that may be palmitoylated.

S1B Four potential cysteine residues were mutated to serine (C8S, C192S, C263S and C278S) though mutating G (left) to C (right) in DNA base and they were all verified by Sanger Sequencing. From the first to the fourth row, the DNA sequences represented VMP1-C8S-Flag, VMP1-C192S-flag, VMP1-C263S-flag, VMP1-C278S-flag respectively. The red boxes displayed where the bases of point mutants were localized.

S1C The expression of different ZDHHCs in Sertoli cells based on the immunohistochemistry results of the Human Protein Atlas.

S1D The knockdown efficacy of each siRNA was verified by real-time RT-PCR.

Supplementary Fig S2A. NTA measurement showed that the sEV amount in shVMP1 group was significantly less than that in the control group (CTR)

S2B. NTA measurement showed oeVMP1 group secreted more sEVs compared with the CTR group

S2C. administration of palmitoylation inhibitor 2BP to oeVMP1 group greatly decreased its sEV release amount to the level that resembled the CTR group

S2D. NTA measurement showed that the sEV release amount in WT group was statistically more than that in MT group and NC group

Supplementary Fig S3A. Representative images showing the colocalization between WT-VMP1/ MT-VMP1 and EEA1-marker for early endosome.

S3B. Representative images showing the colocalization between WT-VMP1/ MT-VMP1 and Rab7b—marker for late endosome.

S3C. HRS expressin (MVB marker) in Sertoli cells overexpressing WT-VMP1 or MT-VMP1.

Supplementary Fig S4A, B. GO and KEGG analyses were performed for WT-VMP1 and MT-VMP1 interacting proteins.

S4C. Proteins present in WT-VMP1 but not in MT-VMP1 and NC groups were for further analysis.

S4D. The knockdown efficiency of siALIX was verified by WB.

Supplementary Fig S5A. In one week old mice, VMP1 was expressed in both SCs and spermatogenic cells

S5B In adult mice (6-week old), VMP1 was mainly expressed in SCs, colocalized with SC marker Vimentin

Supplementary Fig. S1A, S1B, S1C , S1D

A

Predicted Sites

	ID	Position	Peptide	Score	Cutoff
1	Q96GC9	8	***MAENGKNCDQRRVAMNKE	0.8381	0.0000
2	Q96GC9	152	IASVTLAAYECNSVNFPEPPY	0.3528	0.0000
3	Q96GC9	168	PEPPYPDQIICPDEEGTEGTI	0.1208	0.0000
4	Q96GC9	192	SIISKVRIEACMWGIGTAIGE	0.7726	0.0000
5	Q96GC9	263	QKVGFFGILACASIPNPLFDL	0.7264	0.0000
6	Q96GC9	278	NPLFDLAGITCGHFLVPFWTF	0.2923	0.0000
7	Q96GC9	377	MFEKLVVVMVCYFILSIINSM	0.5807	0.0000

B

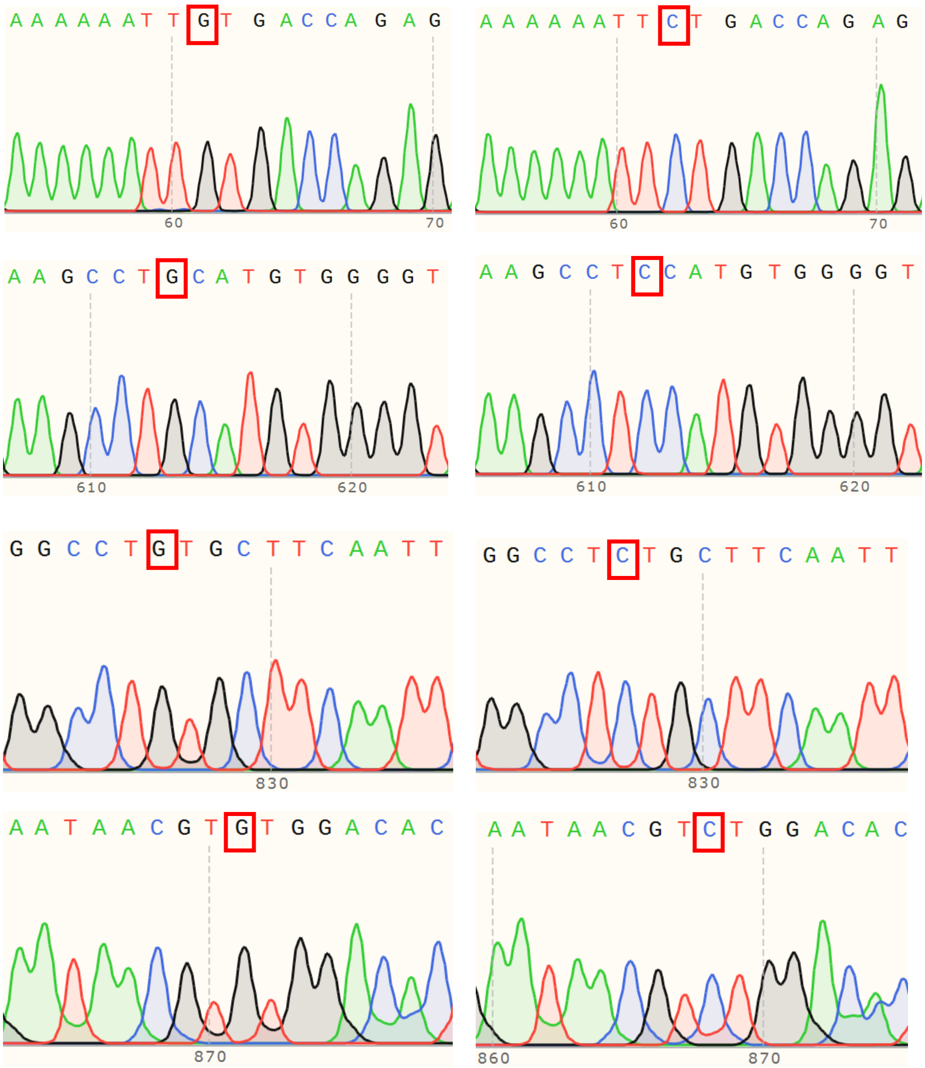

C

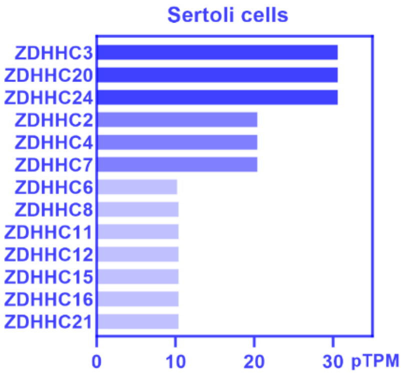

D

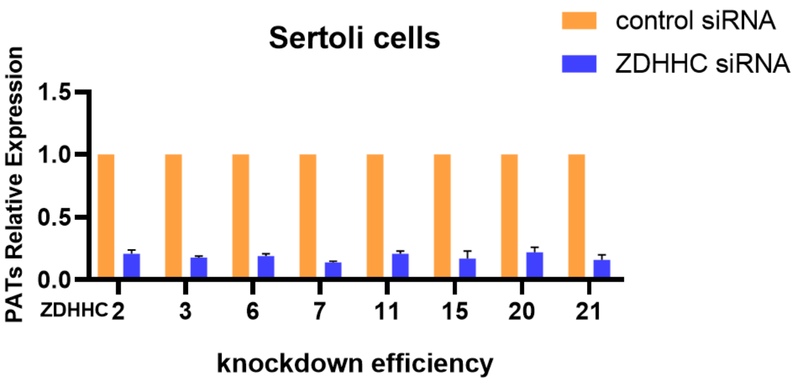

Supplementary Fig. S2A, S2B, S2C, S2D

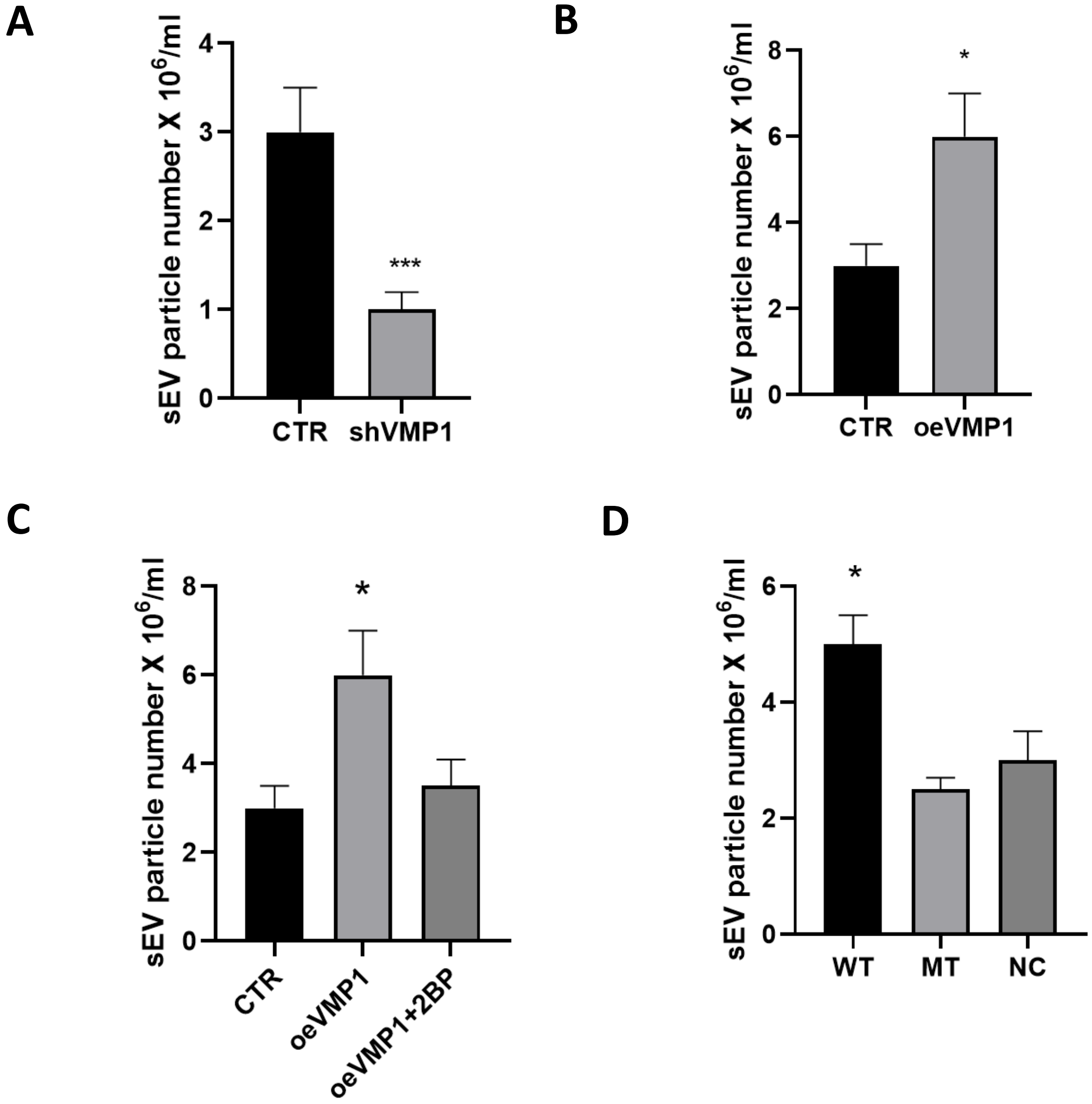

Supplementary Fig. S3A, S3B, S3C

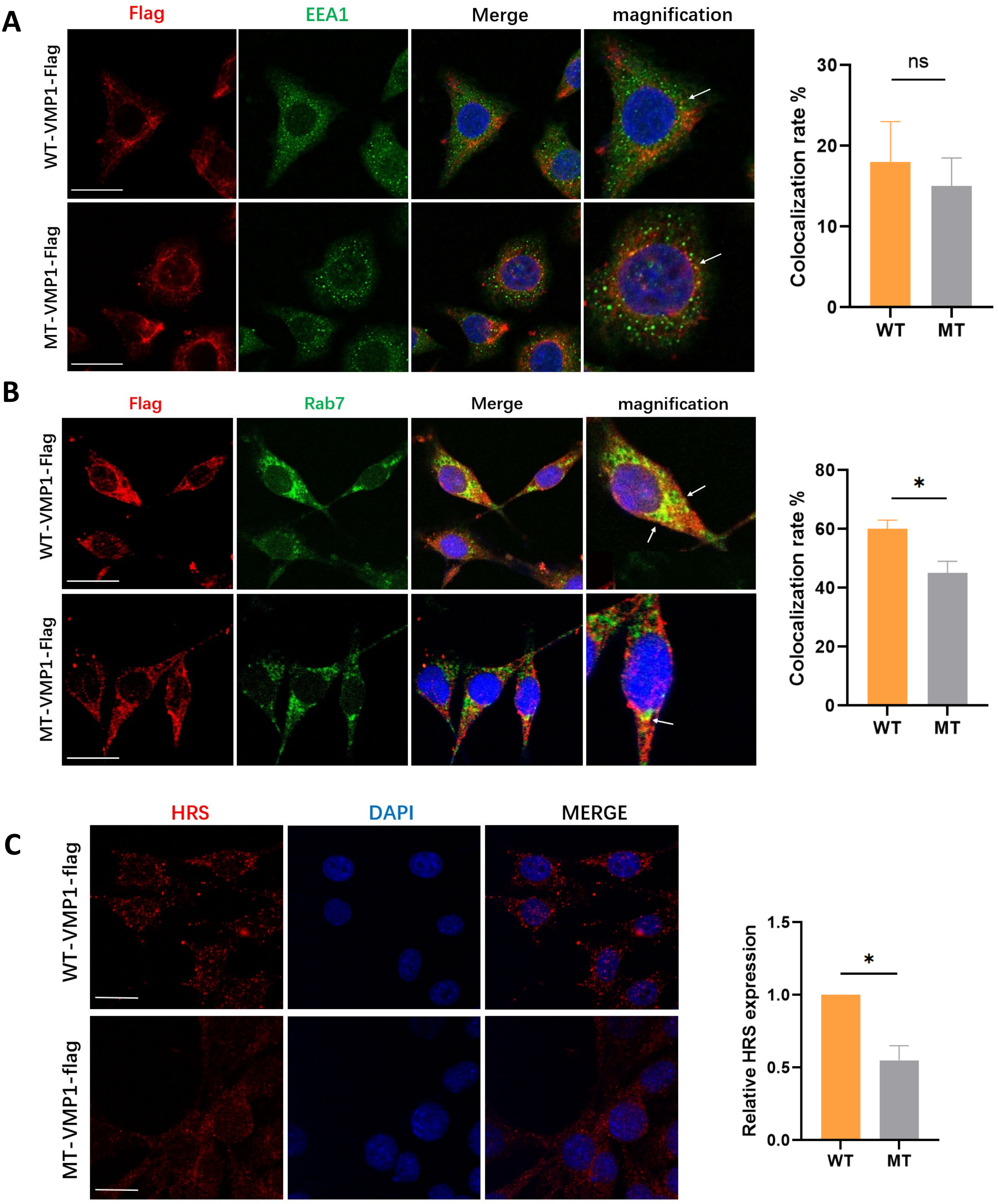

Supplementary Fig. S4A, S4B, S4C, S4D

A

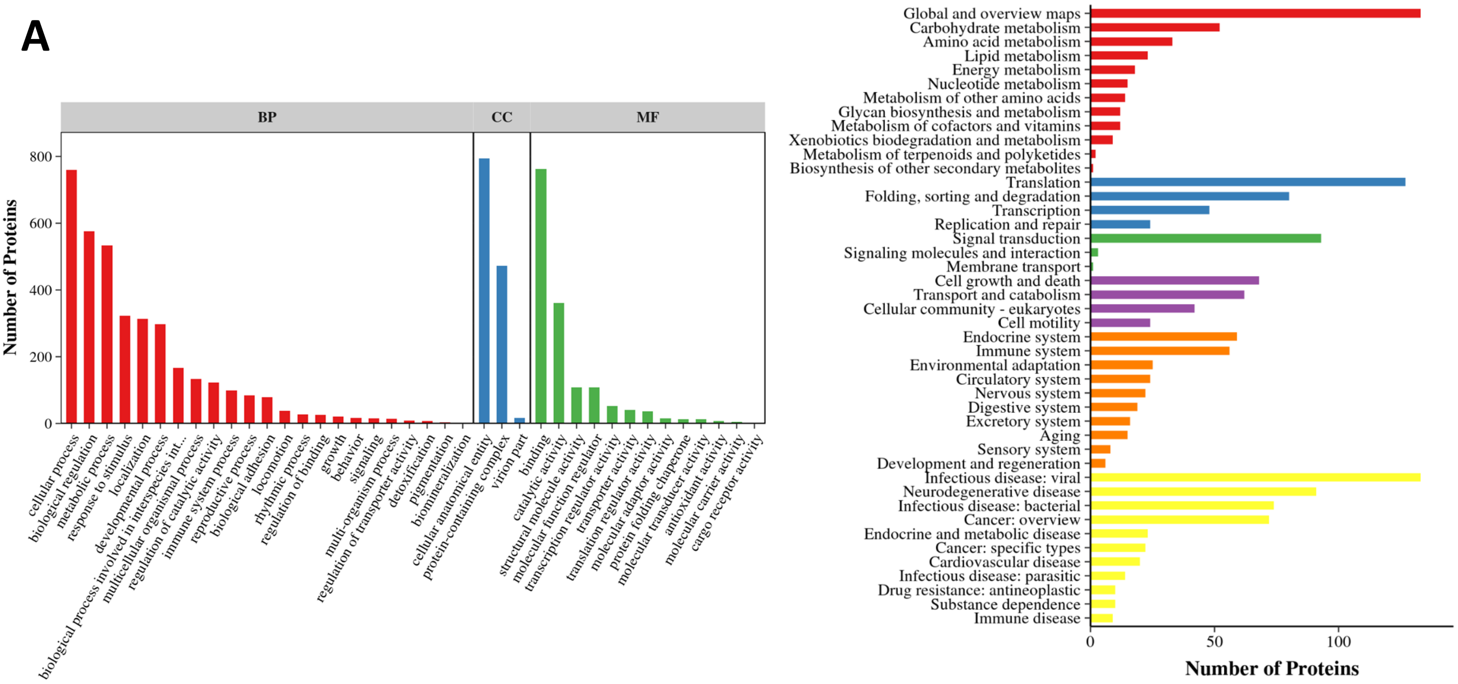

B

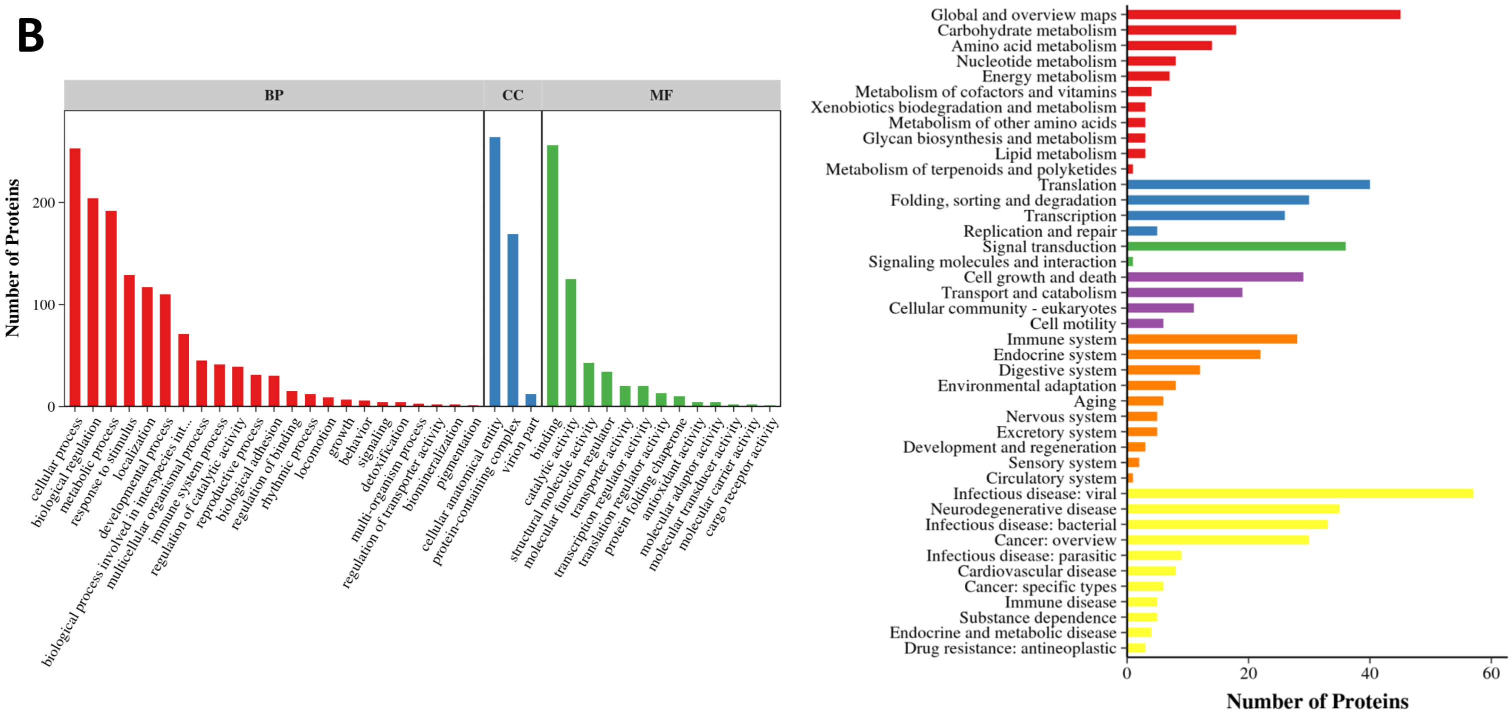

C

Proteins	Major function
RAB1B	COPII vesicle coating, ER to Golgi vesicle-mediated transport
RAB9A	transport of proteins between the endosomes and the trans-Golgi network
RAB32	vesicle-mediated transport, GTP-binding, Nucleotide-binding
COPB2	ER-Golgi transport, Protein transport, Golgi non-clathrin-coated vesicles
AP1B1	Subunit of clathrin-associated adaptor protein complex 1, protein sorting
BCAP31	Chaperone, export of secreted proteins in the ER, ER-Golgi transport
SEC23B	vesicle-mediated transport, Component of the coat protein complex II (COPII)
SNX6	intracellular protein transport, phosphatidylinositol binding
KIF5B	ATPase activity, positive regulation of intracellular protein transport
USO1	ER-Golgi transport, Protein transport, COPII vesicle coating
SNX2	early endosome to Golgi transport, intracellular protein transport
PDCD6IP	endocytosis, MVB biogenesis, membrane repair, cytokinesis, apoptosis

D

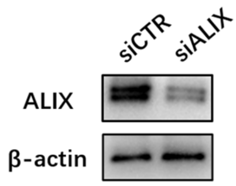

Supplementary Fig. S5A, S5B

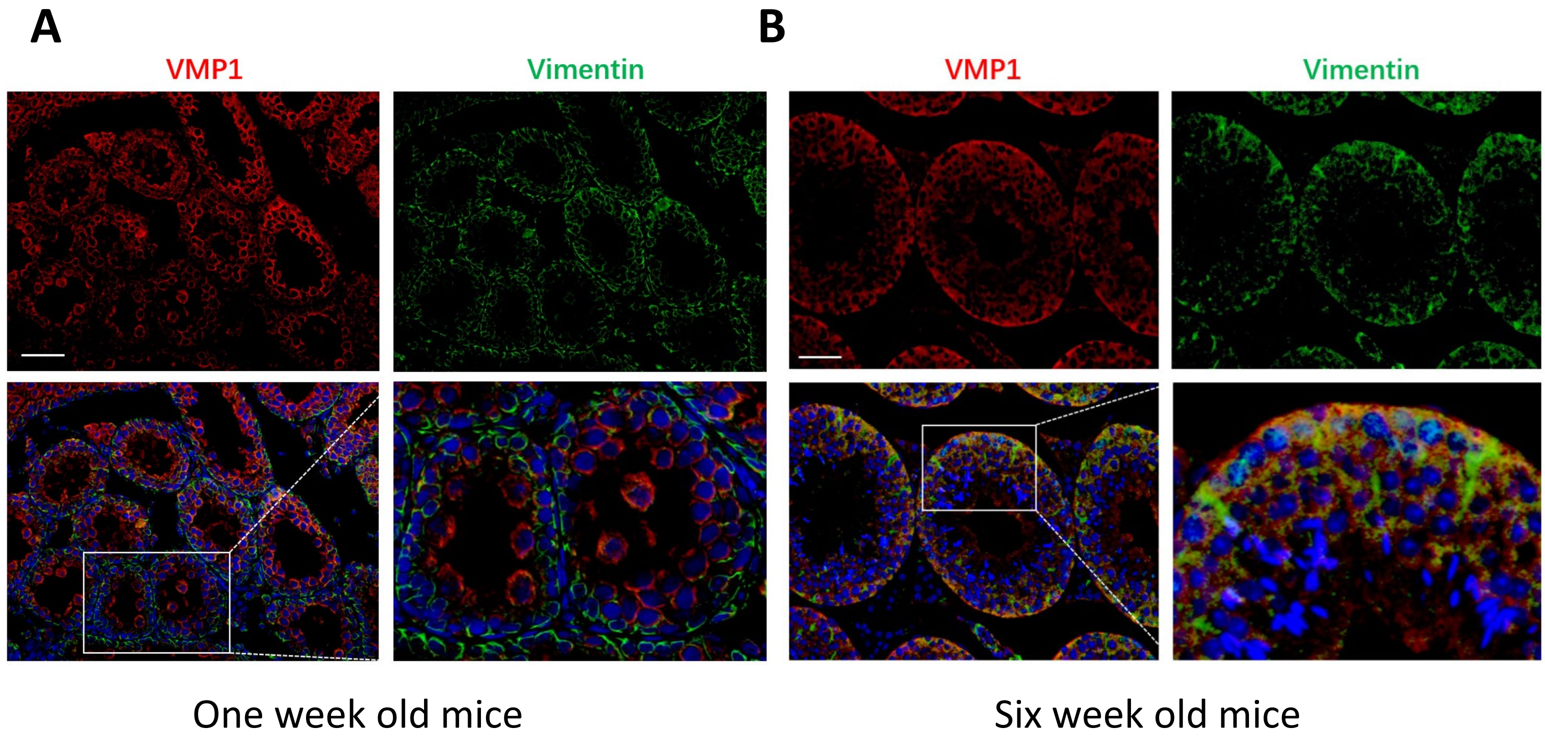

Supplement: Supplementary file 1 — Supplementary Material 1. [file 12964_2024_1529_MOESM1_ESM.pdf]
