# Supplementary material for: Palmitoylation of vacuole membrane protein 1 promotes small extracellular vesicle secretion via interaction with ALIX and influences intercellular communication
Source: Cell Commun Signal. 2024 Feb 26;22:150. doi: 10.1186/s12964-024-01529-6 (PMC10895845; doi:10.1186/s12964-024-01529-6)

original western blots

Fig 1A

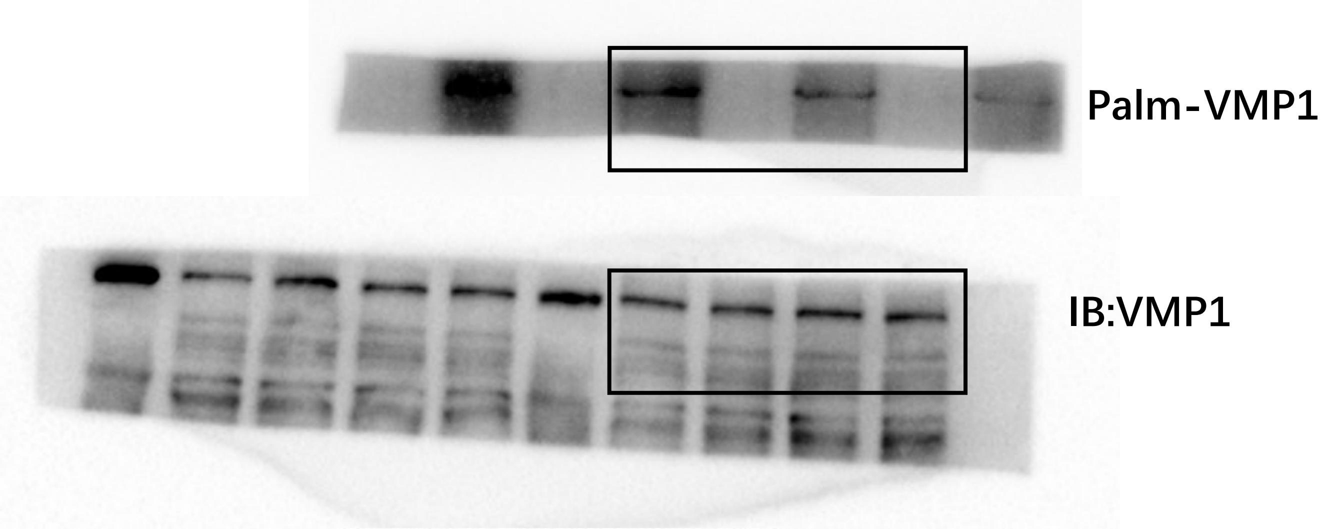

Fig 1B

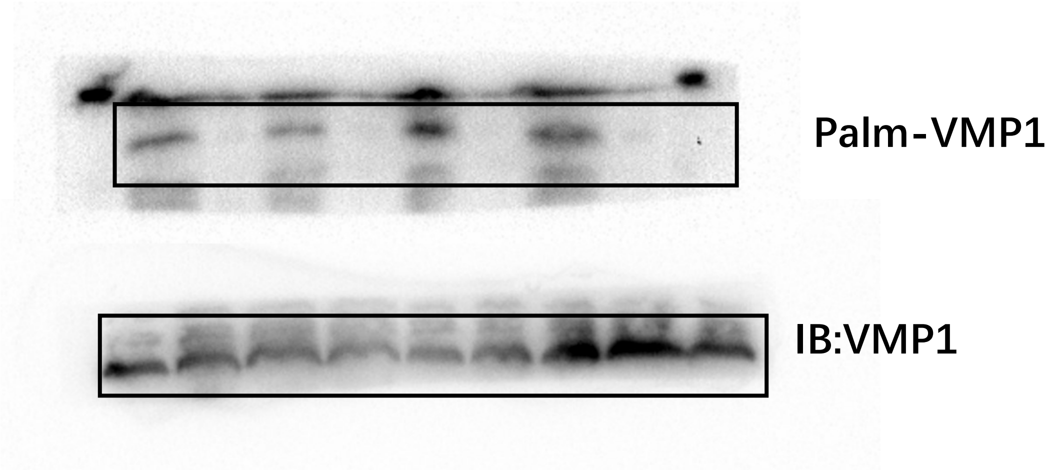

Fig 1E,1F

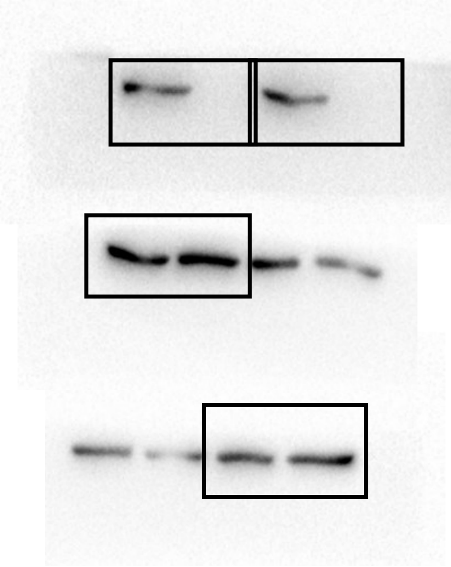

Fig 1G

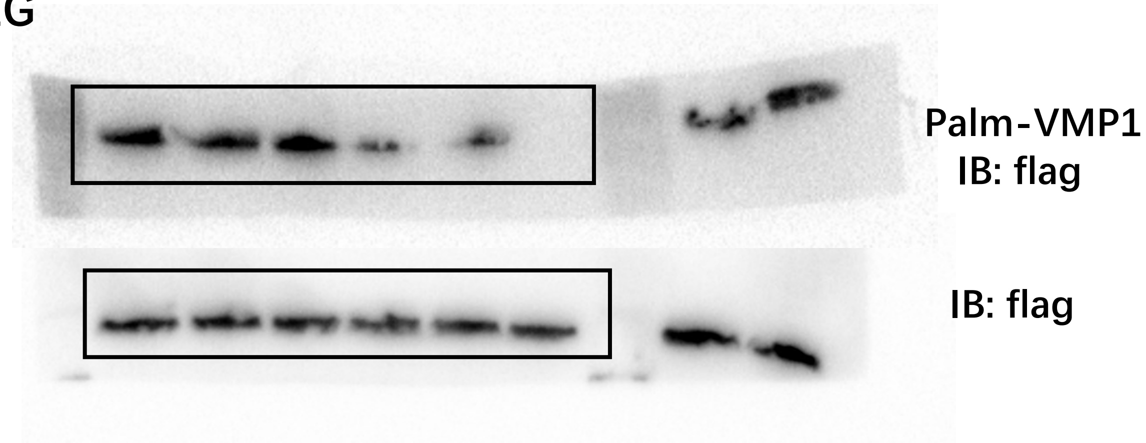

Fig 1H

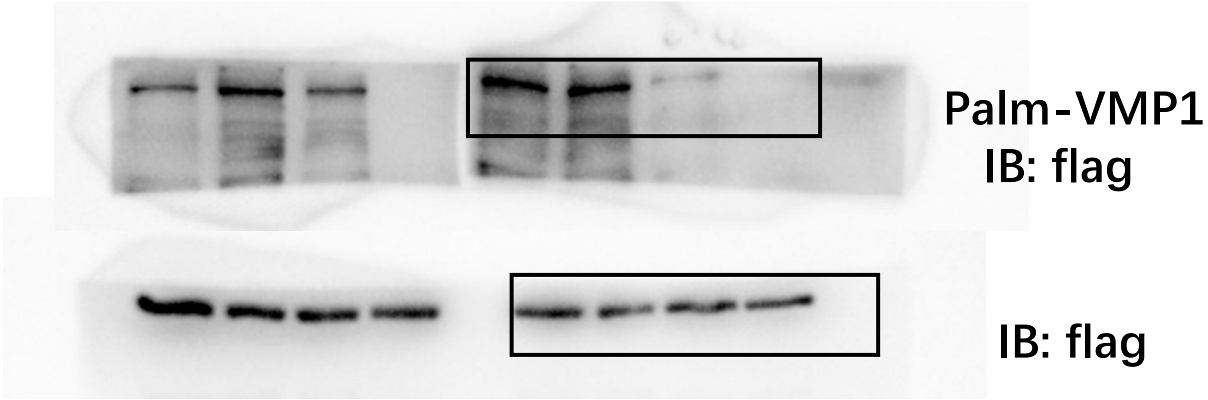

Fig 1K

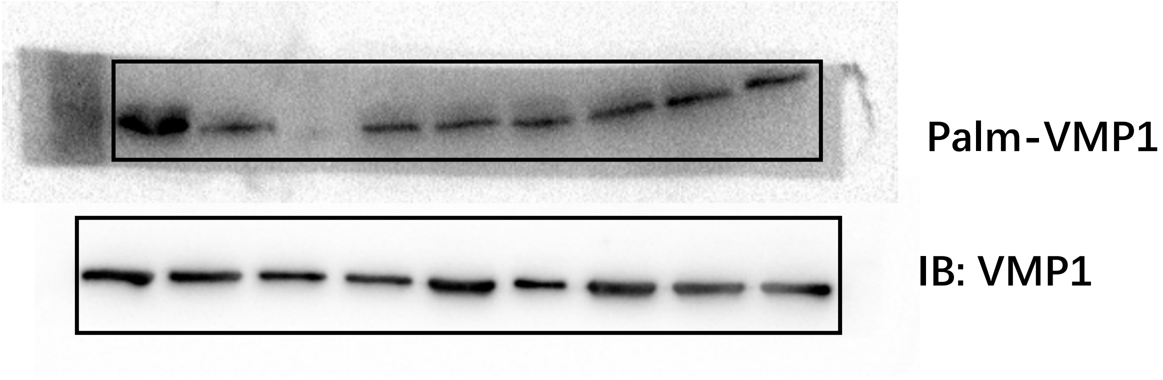

Fig 1L

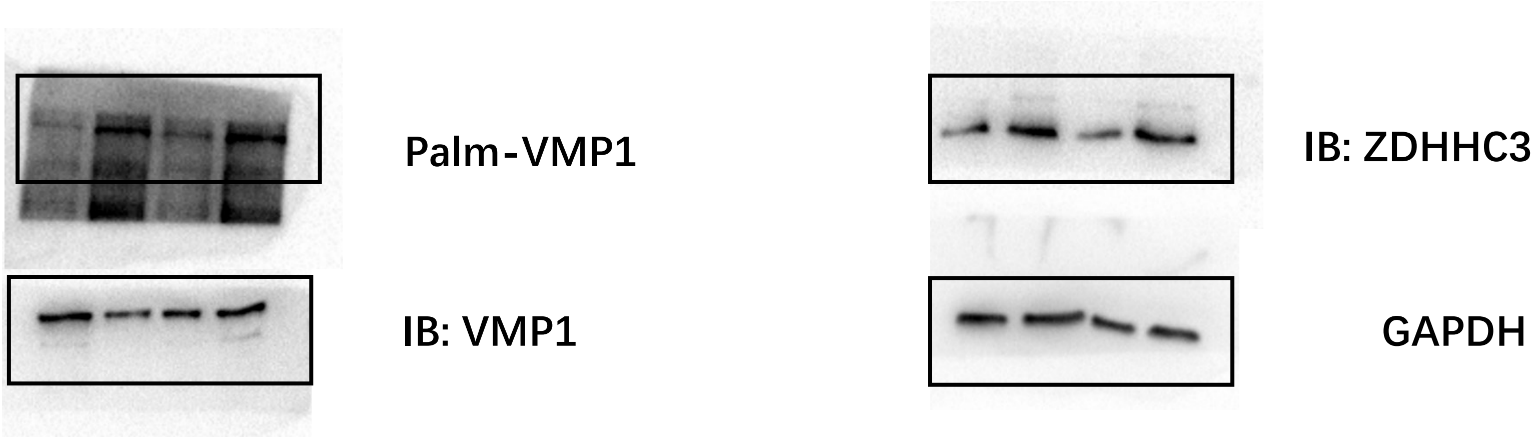

Fig 2A

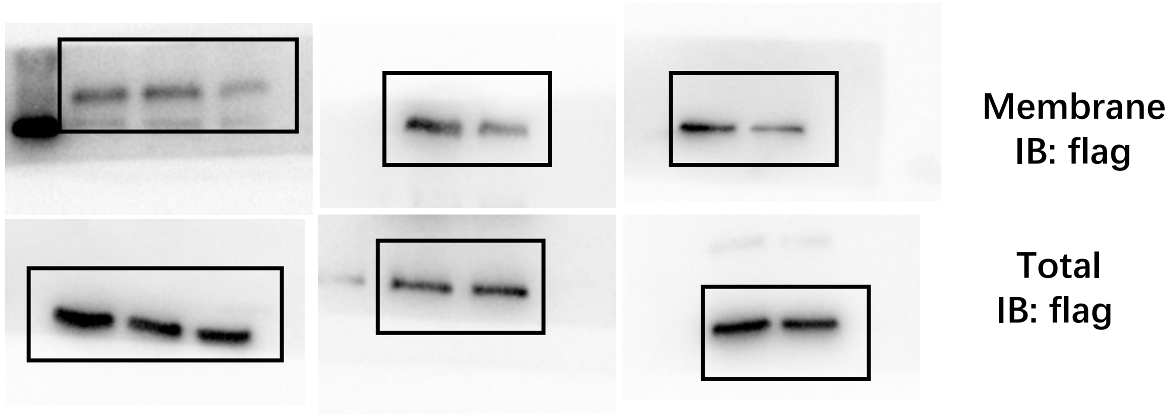

Fig 3C

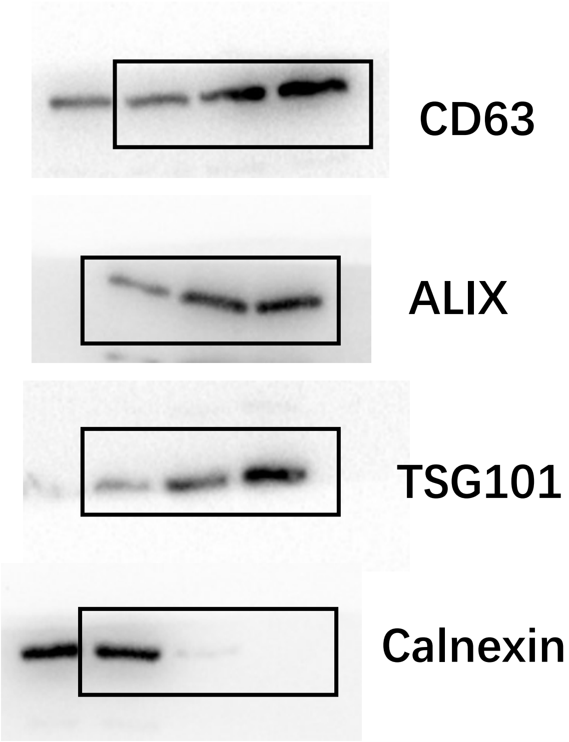

Fig 3D

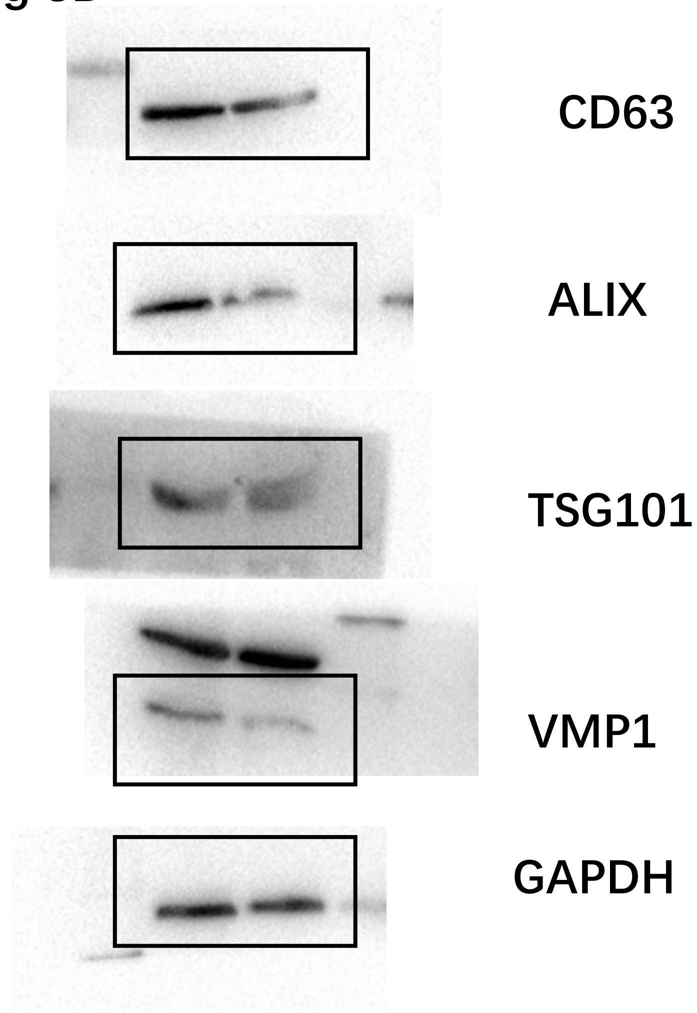

Fig 3E

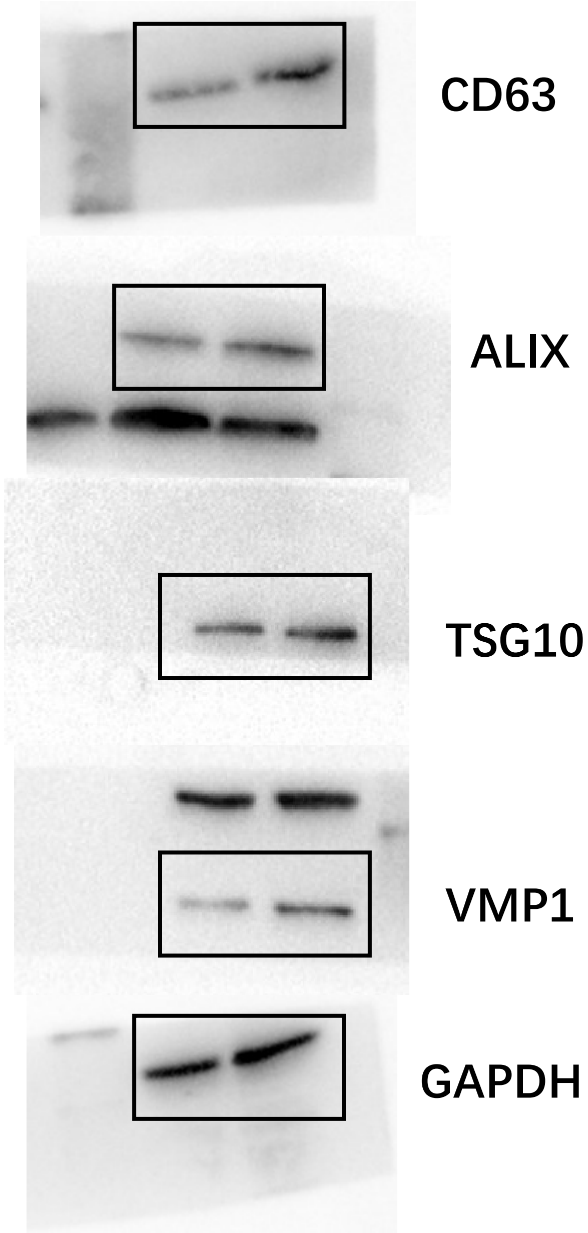
CD63
ALIX
TSG101
VMP1
GAPDH

Fig 3F

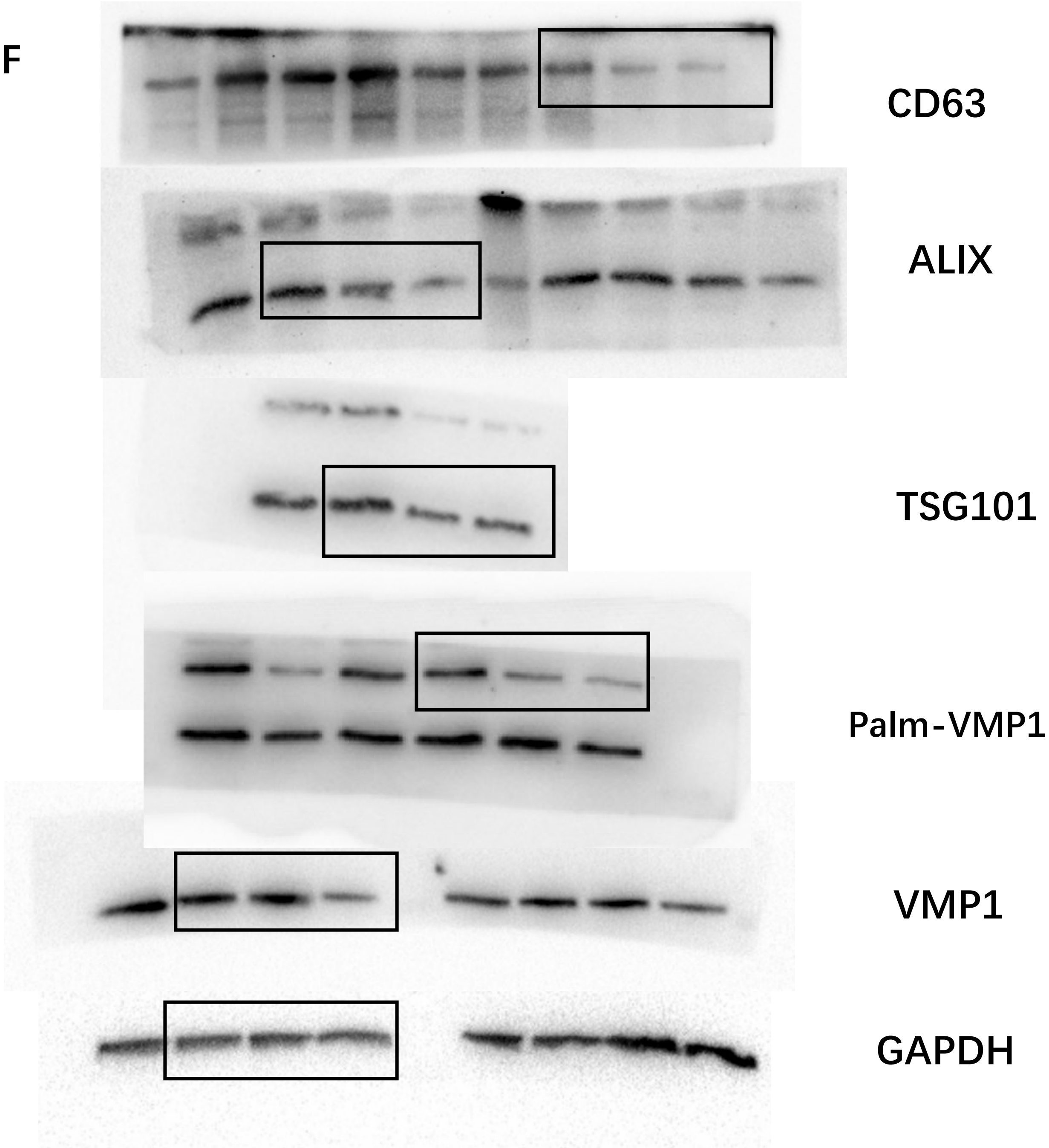
CD63
ALIX
TSG101
Palm-VMP1
VMP1
GAPDH

Fig 5D

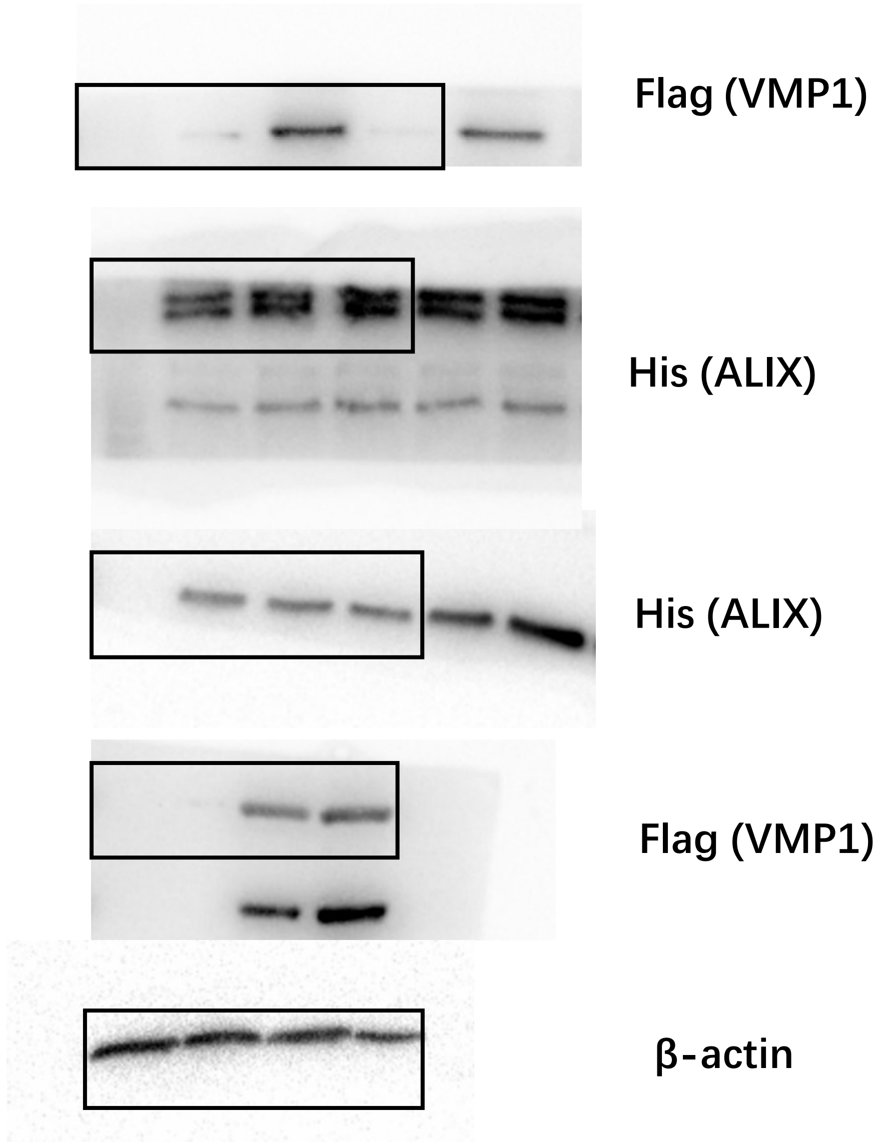
Flag (VMP1)
His (ALIX)
His (ALIX)
Flag (VMP1)
β-actin

Fig 5E

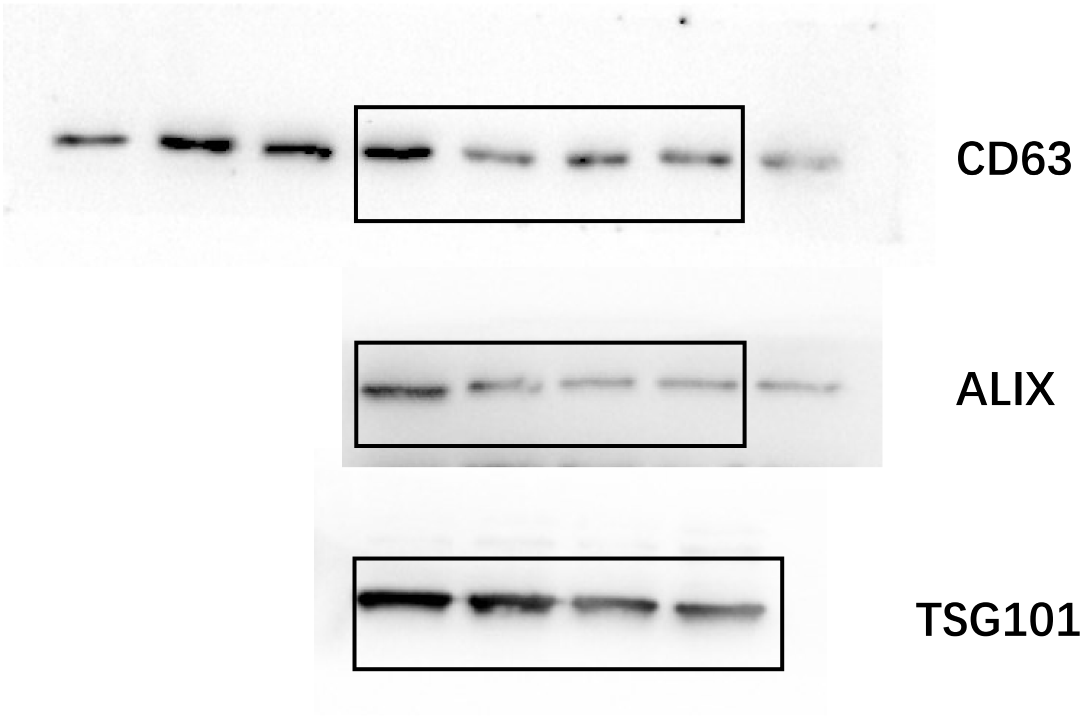
CD63
ALIX
TSG101

Fig 7B

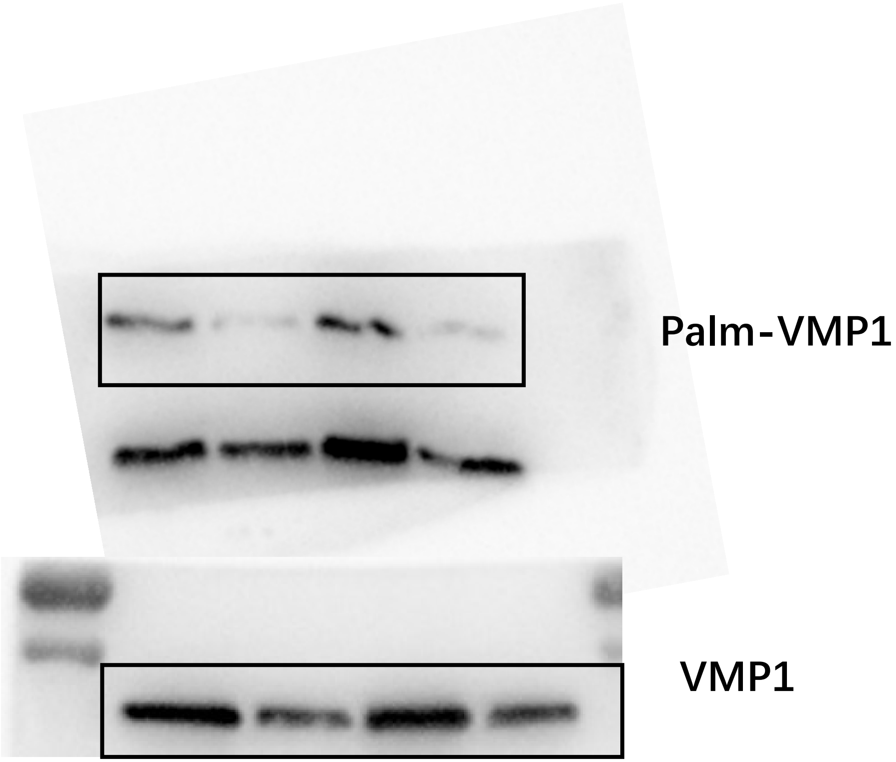

Supplement: Supplementary file 2 — Supplementary Material 2. [file 12964_2024_1529_MOESM2_ESM.pdf]
